# Global Insights into Chronic Obstructive Pulmonary Disease and Coronary Artery Disease: A Systematic Review and Meta-Analysis of 6,400,000 Patients

**DOI:** 10.31083/j.rcm2501025

**Published:** 2024-01-15

**Authors:** Yitian Zheng, Zhenliang Hu, Samuel Seery, Chen Li, Jie Yang, Wenyao Wang, Yu Qi, Chunli Shao, Yi Fu, Han Xiao, Yi-Da Tang

**Affiliations:** ^1^Department of Cardiology and Institute of Vascular Medicine, Peking University Third Hospital; NHC Key Laboratory of Cardiovascular Molecular Biology and Regulatory Peptides; Key Laboratory of Molecular Cardiovascular Science, Ministry of Education; Beijing Key Laboratory of Cardiovascular Receptors Research, 100191 Beijing, China; ^2^Research Unit of Medical Science Research Management/Basic and Clinical Research of Metabolic Cardiovascular Diseases, Chinese Academy of Medical Sciences, 100021 Beijing, China; ^3^State Key Laboratory of Cardiovascular Disease, Fuwai Hospital, National Center for Cardiovascular Diseases, Chinese Academy of Medical Sciences and Peking Union Medical College, 100005 Beijing, China; ^4^School of Humanities and Social Sciences, Chinese Academy of Medical Science & Peking Union Medical College, 100005 Beijing, China; ^5^Faculty of Health and Medicine, Division of Health Research, Lancaster University, LA1 4YW Lancaster, UK; ^6^Department of Physiology and Pathophysiology, School of Basic Medical Sciences, Peking University; Key Laboratory of Molecular Cardiovascular Science, Ministry of Education, 100191 Beijing, China

**Keywords:** chronic obstructive pulmonary disease, coronary artery disease, meta-analysis

## Abstract

**Background:**

The high prevalence of chronic obstructive pulmonary disease (COPD) in coronary artery disease (CAD) has been acknowledged over the past decade, although the cause/s remain uncertain due to differences in diagnoses. COPD has also become a leading CAD comorbidity, although again little is known about its interactions. This meta-analysis explored COPD prevalence in the global CAD population, as well as the influence of COPD on CAD.

**Methods:**

PubMed, Web of Science, Embase, and grey literature were searched until 26th November 2021. The prevalence of COPD was calculated, and data were grouped according to COPD diagnostic methods, interventions, region, economic status, etc. Outcomes including all-cause death, cardiac death, myocardial infarction, revascularization, stroke, heart failure, and respiratory failure were analyzed. This study was registered with PROSPERO (CRD No.42021293270).

**Results:**

There was an average prevalence of 14.2% for COPD in CAD patients (95% CI: 13.3–15.1), with diagnostics of COPD through spirometry, International Classification of the Diseases (ICD codes), and self-reported methods. Comorbid COPD–CAD patients were more likely to be smokers and suffer from cardiovascular and respiratory complications (all odds ratios [OR] >1). COPD–CAD has higher mortality (hazard ratio [HR] 2.81, 95% CI: 2.40–3.29), and myocardial infarction, stroke, and respiratory failure rates (all HR >1). Coronary artery bypass graft (CABG) reduces the need for revascularization (HR 0.43, 95% CI: 0.20–0.94) compared to percutaneous coronary intervention (PCI), without increasing mortality.

**Conclusions:**

The global prevalence of COPD is particularly high in CAD patients. COPD–CAD patients are more likely to encounter cardiovascular and respiratory complications and endure poorer outcomes. Limited evidence suggests that CABG may reduce the need for revascularization without increasing mortality, although further research is required to confirm these observations.

## 1. Introduction

Chronic obstructive pulmonary disease (COPD) is a leading cause of death and incidence increases with age [1]. The number of people with chronic respiratory diseases is estimated to be approximately 544.9 million, with almost 55% experiencing COPD [2]. Likewise, coronary artery disease (CAD) is a leading cause of death and consists of common risk factors, including smoking, pollution, unhealthy diet, as well as genetic variances. The coexistence of COPD and CAD is thought to be common and has a hugely detrimental impact on comorbidity outcomes [3]. Indeed, COPD, as a comorbidity of CAD patients, is receiving increased attention, however, there is currently no systematic review or meta-analysis on this growing trend.

The occurrence of CAD with COPD can be understood from both a physiological perspective, including inflammation activation, hypoxia stress, etc., and by considering common risk factors, such as tobacco use, and aging. de Miguel-Díez *et al*. [4]. reported on the prevalence of COPD in participants who received a percutaneous coronary intervention (PCI) and found that it gradually increased from 6.2% in 2001 to 7.4% in 2011. This highlights a rising global trend of COPD occurring in CAD patients [4, 5]. We know that the prevalence of COPD in the CAD population varies according to diagnostic methods, ethnic differences, and according to socioeconomic differences. Furthermore, some COPD–CAD patients acquired severe dyspnea, hypoxia, and exercise intolerability, which are associated with increased mortality [6]; however, COPD–CAD outcomes vary substantially.

While we are aware that mortality increases with comorbid COPD–CAD and other related outcomes, such as major adverse cardiovascular events (MACEs), revascularization, myocardial infarction (MI), and stroke, there is conflicting evidence. This means that clinical choices related to revascularization for COPD patients have a direct impact and fuel the debate around the most effective intervention—coronary artery bypass graft (CABG) or PCI. Clearly, there is a need to systematically assess the available evidence to identify gaps in our knowledge and recommend further research. Therefore, we conducted this first systematic review and meta-analysis to investigate the prevalence of COPD in CAD patients, as well as to understand how COPD influences CAD.

## 2. Methods 

### 2.1 Search Strategy and Selection Criteria

Search strategies were developed after a discussion with two physicians and a clinical epidemiologist (YDT, CLS, and SS). PubMed, Embase, Web of Science, and grey literature sources were searched exhaustively. Two additional websites, e.g., Chest, and the European Heart Journal, were searched to ensure that all current research was included and because of their respective high impact in publishing circulatory and respiratory systems research. A detailed outline of our search strategy has been provided in the **Supplementary Materials**, as **Supplementary Material 1**.

**Fig. 1.**
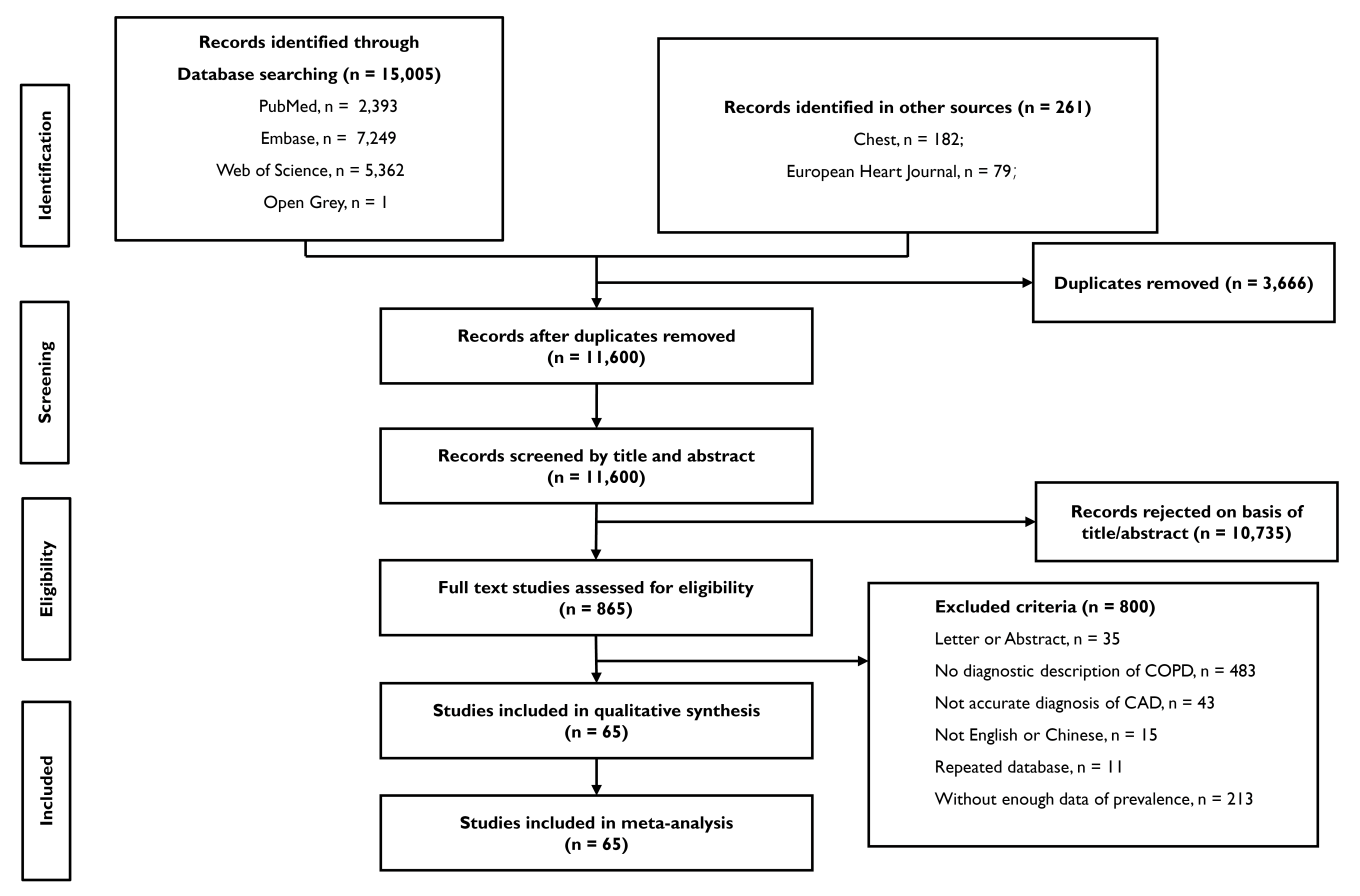
**Flowchart according to PRISMA statement**. Flowchart of the study. COPD, chronic obstructive pulmonary disease; CAD, coronary artery disease.

Studies identified through the aforementioned databases from inception until 26 November 2021 were initially considered eligible. Eligibility criteria are provided in Fig. 1. CAD was diagnosed and included: (1) existing myocardial infarction; (2) those treated with PCI or a CABG; (3) >50% stenosis of at least one of the three major coronary arteries (i.e., left anterior descending, circumflex, or right coronary artery), as observed through coronary angiography.

COPD was diagnosed and classified according to the pulmonary function test (PFT), International Classification of the Diseases (ICD codes), or through self-reported methods. It is important to note that in most studies, the PFT criteria met the gold standard criteria, although a small number of studies involved various other PFT criteria, which were developed before the gold standard was established. ICD codes indicate that patients might have been diagnosed with COPD prior to admission and should not undergo the PFT. Various studies exhibited divergent self-reported methodologies–for instance, a combination of clinical symptoms and COPD medication usage. Studies that did not report diagnostic methods for either CAD or COPD were excluded. Two reviewers independently screened studies (YTZ and ZLH) and discrepancies were resolved by the third reviewer (SS).

Two reviewers (YTZ and ZLH) independently assessed the risk of bias using two separate tools. For the prevalence of COPD–CAD, we used a customized Newcastle–Ottawa Scale (NOS), to classify studies. Scores ≤3 were categorized as high-risk. For outcomes according to COPD status in CAD, we used another customized NOS tool related to outcomes, for which a score ≤6 was thought to indicate a study with a high risk of bias [7]. Details of the customized tools have been provided in **Supplementary Material 2**.

Two reviewers (YTZ and ZLH) extracted and cross-checked data from studies, including demographics and study designs, such as country or region, study type, age, gender, etc. Detailed information has been provided in Table 1 (Ref. [4, 8, 9, 10, 11, 12, 13, 14, 15, 16, 17, 18, 19, 20, 21, 22, 23, 24, 25, 26, 27, 28, 29, 30, 31, 32, 33, 34, 35, 36, 37, 38, 39, 40, 41, 42, 43, 44, 45, 46, 47, 48, 49, 50, 51, 52, 53, 54, 55, 56, 57, 58, 59, 60, 61,62, 63, 64, 65, 66, 67, 68, 69, 70, 71]) and **Supplementary Table 1**. **Supplementary Material 3 **including all supplemental figures and tables.

**Table 1.** **Studies research characteristics**.

Study year (name of the first author/year)	Research held country or region	Area	Economic status	Study type	Patients characteristics	Diagnosed method
Erdil *et al*. 2016 [13]	Turkey	Europe	Upper-middle income	Observational, single center	CAD patients underwent CABG	Pulmonary function test
Geçmen *et al*. 2016 [14]	Turkey	Europe	Upper-middle income	Observational, single center	CAD patients underwent CABG	Pulmonary function test
Barandon *et al*. 2008 [15]	France	Europe	High income	Observational, single center	CAD patients underwent CABG	Pulmonary function test
Yangui *et al*. 2021 [16]	Tunisia	Africa	Upper-middle income	Observational, single center	CAD patients	Pulmonary function test
Almagro *et al*. 2015 [17]	Spain	Europe	High income	Observational, single center	CAD patients underwent PCI	Pulmonary function test
Campo *et al*. 2016 [18]	Italy	Europe	High income	Observational, single center	MI patients with smoking	Pulmonary function test
Stelle *et al*. 2011 [19]	United States of America	North America	High income	Observational, single center	CAD patients underwent CABG	Pulmonary function test
Hamrah *et al*. 2015 [20]	Japan	Asia	High income	Observational, single center	CAD patients	Pulmonary function test
Dagenais *et al*. 2010 [21]	Canada	North America	High income	Observational, single center	CAD patients over 70 years old, who underwent CABG	Pulmonary function test
Komaru *et al*. 2017 [22]	Japan	Asia	High income	Observational, single center	CAD patients	Pulmonary function test
Khassawneh *et al*. 2018 [23]	Jordan	Asia	Upper-middle income	Observational, single center	CAD patients	Pulmonary function test
Ovalı *et al*. 2018 [24]	Turkey	Europe	Upper-middle income	Observational, single center	CAD patients underwent CABG	Pulmonary function test
Çağdaş *et al*. 2019 [25]	Turkey	Europe	Upper-middle income	Observational, single center	CAD patients underwent PCI	Self-reported method
Soliman Hamad *et al*. 2011 [26]	Netherlands	Europe	High income	Observational, single center	CAD patients underwent CABG with EF <30%	Self-reported method
Vlahou *et al*. 2016 [27]	Greece	Europe	High income	Observational, single center	CAD patients underwent CABG	Pulmonary function test
Ponomarev *et al*. 2017 [28]	Russia	Europe	High income	Observational, single center	CAD patients underwent CABG	Pulmonary function test
Ko *et al*. 2016 [29]	China	Asia	Upper-middle income	Observational, single center	CAD patients underwent PCI	Pulmonary function test
Kuo *et al*. 2016 [30]	Taiwan region	Asia	High income	Administrative database	MI patients	ICD codes
Schachner *et al*. 2005 [31]	Austria	Europe	High income	Observational, single center	CAD patients underwent CABG	Self-reported method
Sá *et al*. 2010 [32]	Brazil	South America	Upper-middle income	Observational, single center	CAD patients underwent CABG	Self-reported method
Topcu *et al*. 2017 [33]	Turkey	Europe	Upper-middle income	Observational, single center	CAD patients	Pulmonary function test
DeRose *et al*. 2005 [34]	United States of America	North America	High income	Observational, single center	CAD patients underwent CABG with EF <25%	Self-reported method
Najafi *et al*. 2015 [35]	Iran	Asia	Upper-middle income	Observational, single center	CAD patients underwent CABG	Pulmonary function test
Șerban *et al*. 2019 [36]	Romania	Europe	Upper-middle income	Observational, single center	MI patients	Self-reported method
Medalion *et al*. 2004 [37]	Israel	Asia	High income	Observational, single center	CAD patients underwent CABG	Self-reported method
Yokoyama *et al*. 2000 [38]	United States of America	North America	High income	Observational, single center	CAD patients underwent CABG	Self-reported method
Lazzeri *et al*. 2013 [39]	Italy	Europe	High income	Observational, single center	MI patients underwent PCI	Self-reported method
Canver *et al*. 1998 [40]	United States of America	North America	High income	Observational, single center	CAD patients underwent CABG	Pulmonary function test
Fuster *et al*. 2006 [41]	Spain	Europe	High income	Administrative database	CAD patients underwent CABG	Pulmonary function test
Cohen *et al*. 1997 [42]	Israel	Asia	High income	Observational, single center	CAD patients underwent CABG	Self-reported method
Oliveira *et al*. 2017 [43]	Brazil	South America	Upper-middle income	Observational, single center	CAD patients underwent CABG	Self-reported method
Prapas *et al*. 2007 [44]	Greece	Europe	High income	Observational, single center	CAD patients underwent CABG	Self-reported method
Wang *et al*. 2021 [9]	Multiple countries	N/A	N/A	Randomized clinical trial	CAD patients underwent revascularization	Self-reported method
Magnuson *et al*. 2013 [45]	Multiple countries	N/A	N/A	Randomized clinical trial	CAD patients with diabetes	ICD codes
Huang *et al*. 2019 [8]	Multiple countries	N/A	N/A	Randomized clinical trial	CAD patients underwent revascularization	Self-reported method
Zhang *et al*. 2016 [46]	China	Asia	Upper-middle income	Observational, single center	CAD patients underwent PCI	Self-reported method
Salisbury *et al*. 2007 [47]	United States of America	North America	High income	Observational, multicenter	MI patients	Self-reported method
Dai-Yin Lu *et al*. 2017 [48]	Taiwan region	Asia	High income	Administrative database	CAD patients underwent CABG	ICD codes
Macchia *et al*. 2008 [49]	Italy	Europe	High income	Administrative database	MI patients	ICD codes
Angouras *et al*. 2010 [50]	Greece	Europe	High income	Observational, single center	CAD patients underwent CABG	Pulmonary function test
Gatta *et al*. 2022 [51]	United Kingdom	Europe	High income	Observational, single center	CAD patients underwent CABG	Pulmonary function test
Çakalağaoğlu *et al*. 2020 [52]	Turkey	Europe	Upper-middle income	Observational, single center	CAD patients underwent CABG	Self-reported method
Berger *et al*. 2004 [53]	United States of America	North America	High income	Observational, multicenter	CAD patients underwent PCI	Pulmonary function test
Jatene *et al*. 2017 [11]	Multiple countries	N/A	N/A	Randomized clinical trial	CAD patients underwent PCI	Self-reported method
Efird *et al*. 2013 [54]	United States of America	North America	High income	Observational, single center	CAD patients underwent CABG	Pulmonary function test
Su *et al*. 2017 [55]	Taiwan region	Asia	High income	Administrative database	MI patients	ICD codes
Maynard *et al*. 2006 [56]	United States of America	North America	High income	Administrative database	MI patients	ICD codes
Clement *et al*. 2020 [57]	United States of America	North America	High income	Administrative database	CAD patients underwent CABG	ICD codes
Nishiyama *et al*. 2010 [58]	Japan	Asia	High income	Observational, multicenter	CAD patients underwent revascularization	Self-reported method
O’Boyle *et al*. 2013 [59]	United Kingdom	Europe	High income	Administrative database	CAD patients underwent CABG	Pulmonary function test
Konecny *et al*. 2010 [60]	United States of America	North America	High income	Observational, single center	CAD patients underwent PCI	ICD codes
Hawkins *et al*. 2009 [61]	Multiple countries	N/A	N/A	Randomized clinical trial	MI patients	Self-reported method
Tomaniak *et al*. 2020 [10]	Multiple countries	N/A	N/A	Randomized clinical trial	CAD patients underwent PCI	Self-reported method
Hong *et al*. 2019 [62]	Canada	North America	High income	Observational, single center	CAD patients	Self-reported method
Butt *et al*. 2019 [63]	Denmark	Europe	High income	Administrative database	CAD patients underwent CABG	ICD codes
Kostis *et al*. 1994 [64]	United States of America	North America	High income	Administrative database	MI patients	ICD codes
Andell *et al*. 2014 [65]	Sweden	Europe	High income	Observational, multicenter	MI patients	ICD codes
Elbaz-Greener *et al*. 2020 [66]	Israel	Asia	High income	Administrative database	MI patients underwent CABG	ICD codes
Deo *et al*. 2021 [67]	United States of America	North America	High income	Administrative database	CAD patients underwent CABG	ICD codes
Lin *et al*. 2019 [12]	Taiwan region	Asia	High income	Administrative database	CAD patients underwent PCI	ICD codes
Sundaram *et al*. 2020 [68]	United Kingdom	Europe	High income	Administrative database	MI patients	Self-reported method
de Miguel-Díez *et al*. 2015 [4]	Spain	Europe	High income	Administrative database	CAD patients underwent revascularization	ICD codes
Krittanawong *et al*. 2020 [69]	United States of America	North America	High income	Administrative database	MI patients <55 years	ICD codes
Johnson-Sasso *et al*. 2018 [70]	United States of America	North America	High income	Administrative database	MI patients	ICD codes
Neumann *et al*. 2020 [71]	Germany	Europe	High income	Administrative database	MI patients	ICD codes

CAD, coronary artery disease; MI, myocardial infarction; CABG, coronary artery bypass graft; PCI, percutaneous coronary intervention; ICD, International Classification of the Diseases; N/A, not applicable; EF, ejection fraction.

### 2.2 Data Analysis

The random effects model (DerSimonian and Laird method) was implemented due to assumed differences within (and between) populations. Estimates with 95% confidence intervals (CI) have been provided. We adopted two types of proportional transformation, i.e., Logit transformation and Freeman–Tukey double arcsine transformation for sensitivity analysis. Leave-one-out analysis and exclusion analysis were conducted according to the number of participants in specific subgroups.

Subgroup analysis of COPD diagnostics, study type, location of study, economic status (according to World Bank), and risk of bias, were performed to identify potential sources of heterogeneity. Univariate meta-regression analyses were performed, with the prevalence of COPD in CAD as the dependent variable.

Independent variables included the COPD definition, economic status, study type, risk of bias, area, age, male, hypertension, diabetes mellitus, dyslipidemia, atrial fibrillation, stroke, and smoker status. Independent variables with *p* < 0.05 were enlisted for multivariate meta-regression analyses. The proportion of variance in prevalence estimates was explained using R square calculations [72, 73].

For comorbidities and risk factors in the COPD group, odds ratios (ORs) with corresponding 95% CIs were calculated according to COPD status. Values with 95% CIs that did not include one were accepted as statistically significant.

For outcomes related to COPD–CAD status, the primary endpoint was all-cause mortality. Secondary endpoints included cardiac death, stroke, revascularization, myocardial infarction (MI), heart failure, and respiratory failure. The random effect model was also implemented to pool a conservative risk ratio of COPD (compared to non-COPD) in CAD, according to various endpoints.

Subgroup analyses were performed to compare mortality in different groups, specifically the PFT versus ICD codes/self-reported method, and CABG vs. PCI. Leave-one-out analysis was again performed to assess the impact of single studies on pooled risk ratios.

Studies that reported comparisons in outcomes related to CABG and PCI in COPD patients were enlisted for meta-analysis. Outcomes related to revascularization methods, such as all-cause death, myocardial infarction, stroke, and revascularization were established as endpoints. Additionally, publication bias was assessed using Egger’s test, with results presented in the form of a funnel plot.

All statistical analyses were performed using Stata (version 13.0, StataCorporation, Austin, TX, USA) and R (version 4.0.5, R Foundation for Statistical Computing, Vienna, Austria). This study was registered with PROSPERO (CRD #42021293270) and in accordance with the Preferred Reporting Items for Systematic Reviews and Meta-Analyses (PRISMA) guidelines. Please see **Supplementary Material 4 **for further details.

## 3. Results

**Fig. 2.**
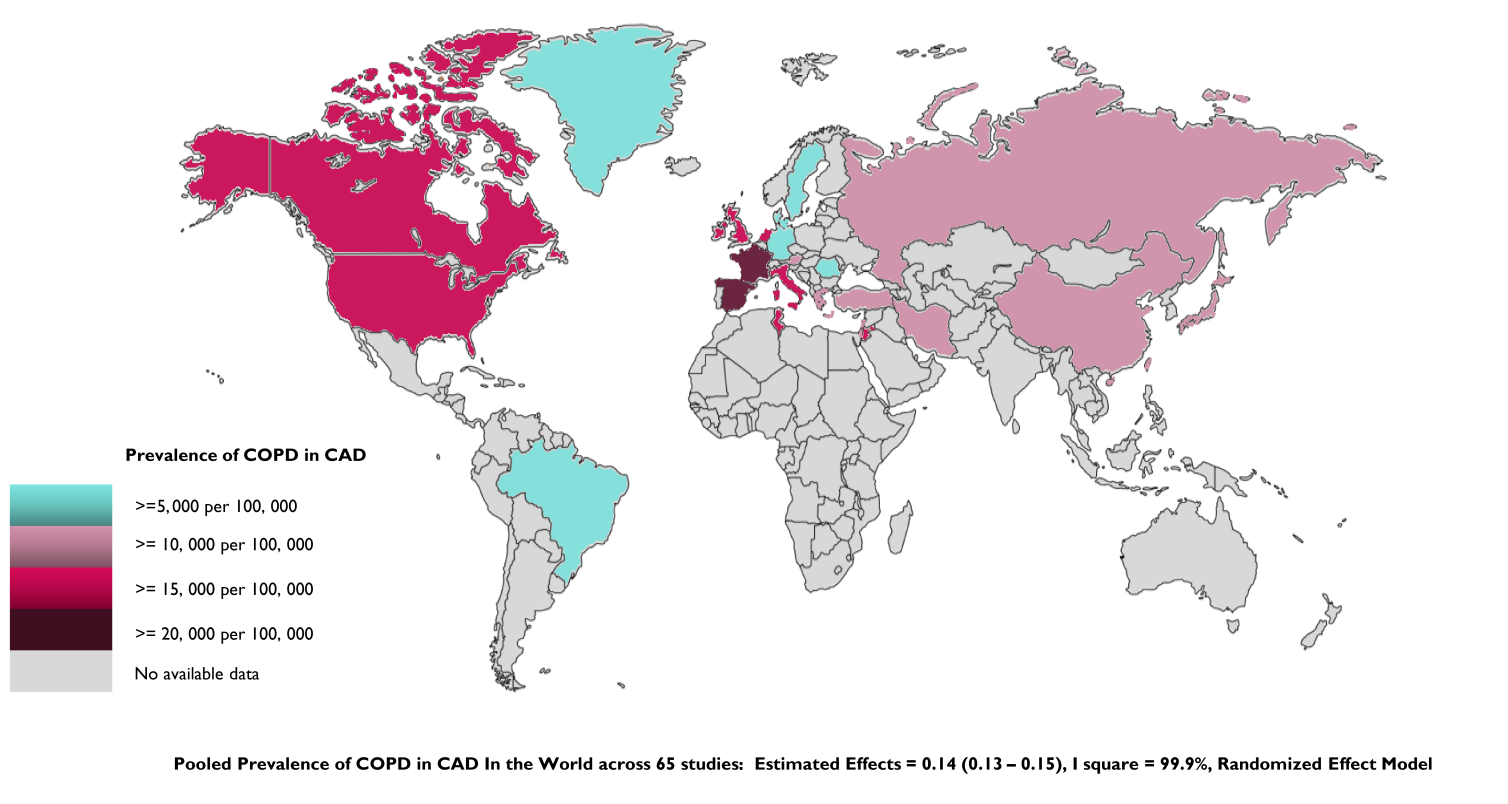
**Global prevalence of COPD in CAD by country**. Countries for which data were unavailable are shown in grey. COPD, chronic obstructive pulmonary disease; CAD, coronary artery disease.

We created a graphical abstract for ease (please see the structural graphical abstract appended). After searching databases and specific websites, we initially identified approximately 15,000 studies. Once duplicates had been excluded, 11,600 study titles were screened. A total of 10,735 studies were excluded at this screening stage, meaning 865 reports remained and the abstracts were read. Sixty-five studies were finally included for a full examination and data were extracted for pooling purposes (Fig. 2).

### 3.1 Global Prevalence and Comorbidities

Study and participant characteristics, such as study type, research location, economic status, etc., are provided in Table 1 and **Supplementary Table 1**. Forest plots suggested that the pooled prevalence of COPD in CAD patients is 14.2% (95% CI: 13.3–15.1). Please see the **Supplementary Materials**, **Supplementary Fig. 1**, for further details. **Supplementary Fig. 2** showed the publication bias of each study.

Sensitivity analysis was conducted using the inverse variance and Logit transformation methods and a similar prevalence was reported for each (**Supplementary Table 2**). Leave-one-out analysis suggested that there was no significant impact by a single study on the pooled COPD–CAD prevalence (**Supplementary Fig. 3**). However, during the sensitivity analysis, and by excluding studies according to sample size, some heterogeneity was found to exist. By initially excluding the smallest sample of studies, we found that heterogeneity was closely related to studies using the PFT as a diagnostic method (**Supplementary Tables 3,4**).

**Fig. 3.**
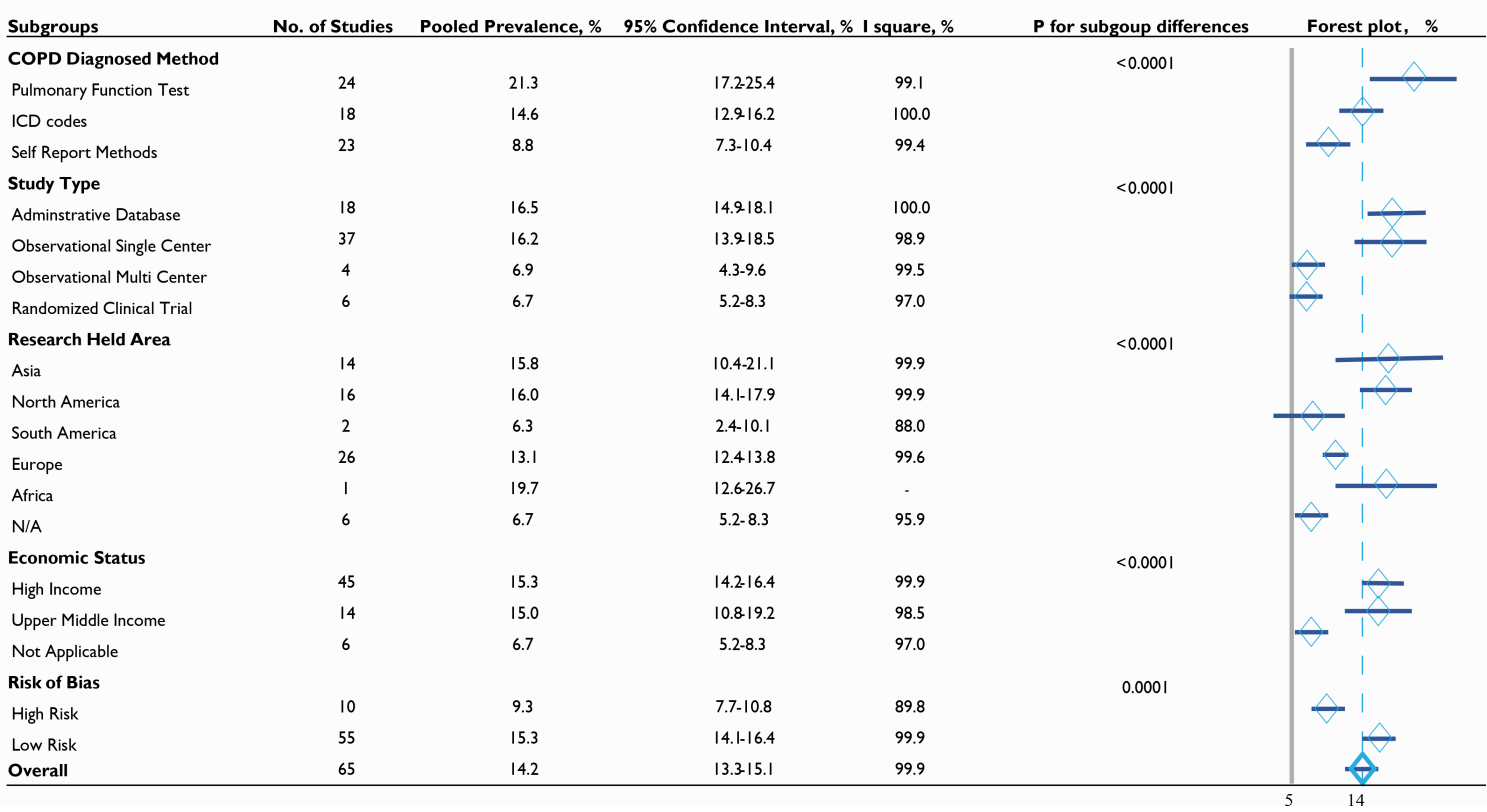
**Prevalence of COPD in CAD in various subgroups**. COPD, chronic obstructive pulmonary disease; ICD, International Classification of the Diseases; N/A, not applicable.

The pooled prevalence of COPD–CAD across different countries or regions was presented as a visualized version of the world map, with different colors indicating the extent of the COPD–CAD prevalence (Fig. 3). From the heat map, one can see that the prevalence appears highest in North America, followed by Asia, Europe, and South America. For countries in Africa and Oceania, evidence of COPD prevalence in CAD is lacking, with only one study from the African continent reporting on prevalence (Fig. 3).

The analysis of subgroups also helped to uncover potential factors that may influence prevalence. For example, the prevalence in the PFT group was significantly higher than the rate observed in the ICD codes group or in the self-reported group (21.3% vs. 14.6% vs. 8.8%) and was also significant (*p* < 0.0001) (Fig. 3). There was no obvious difference between high-income countries and upper-middle-income countries, although there was statistical significance when comparing these with “undetermined income” countries, which included multinational clinical trials (15.3% vs. 15.0% vs. 6.7%, *p* < 0.0001).

Univariate and multivariate meta-regression analyses were performed to identify potential sources of heterogeneity. COPD diagnostics appears to be the main source of heterogeneity, followed by study type, economic status, and diabetes mellitus (all *p* < 0.05; $R^{2}$: 28.39% vs. 15.01% vs. 6.32% vs. 5.76%). See **Supplementary Table 3** for details. After imputing these factors into the multivariate model, statistical significance (*p* = 0.0016; $R^{2}$ = 23.64%) remained (**Supplementary Table 5**).

Information on OR related to patient characteristics, according to COPD status, is provided in Table 2. A total of 23 studies reported the number of men in the COPD group and non-COPD groups, with no obvious differences noted according to gender (OR = 1.001, 95% CI: 0.87–1.15). A total of 17 studies also reported dyslipidemia rates in the two groups, although, again, no statistically significant differences were observed (OR = 1.03, 95% CI: 0.89–1.19).

**Table 2.** **Odds ratios related to patient characteristics according to COPD in CAD**.

Variables		No. of studies	OR	Tau square for OR	I square for OR, %	Pooled COPD prevalence in CAD (95% CI), %
Smoker		22	1.94 (1.57–2.40)	0.211	96.0	17.0 (14.7–19.3)
	sub: non-smoker		-	-	-	10.5 (9.0–11.9)
Hypertension		25	1.36 (1.20–1.53)	0.070	92.7	14.5 (12.4–16.6)
	sub: non-hypertension		-	-	-	10.6 (9.4–11.8)
Diabetes mellitus		25	1.18 (1.10–1.27)	0.016	76.5	14.6 (12.6–16.7)
	sub: non-DM		-	-	-	13.0 (11.5–14.5)
Dyslipidemia		17	1.03 (0.89–1.19)	0.068	93.0	14.3 (11.8–16.8)
	sub: non-dyslipidemia		-	-	-	13.9 (11.6–16.3)
Atrial fibrillation		8	1.64 (1.14–2.36)	0.169	79.1	30.3 (17.3–43.3)
	sub: non-AF		-	-	-	17.1 (11.1–23.1)
Stroke		13	1.72 (1.35–2.18)	0.143	95.3	18.8 (14.9–22.7)
	sub: non-Stroke		-	-	-	12.5 (10.9–14.1)
Male		23	1.00 (0.87–1.15)	0.089	95.0	13.7 (12.0–15.3)
	sub: female		-	-	-	12.7 (10.9–14.5)
Dyspnea		4	4.11 (2.65–6.38)	0.084	36.7	29.6 (18.7–40.5)
	sub: non-dyspnea		-	-	-	5.9 (2.1–9.7)
Wheezes		2	9.86 (1.08–90.20)	2.021	75.7	69.7 (16.7–122.7)
	sub: non-wheezing		-	-	-	11.7 (7.1–16.2)
Chronic bronchitis		2	19.07 (5.14–70.81)	0.505	43.8	67.3 (24.3–110.3)
	sub: non-chronic bronchitis		-	-	-	9.3 (2.4–16.1)

COPD, chronic obstructive pulmonary disease; CAD, coronary artery disease; OR, odds ratio; CI, confidence interval; AF, atrial fibrillation; DM, diabetes mellitus.

Further comorbidities and risk factors, including hypertension, diabetes mellitus, atrial fibrillation, stroke, smoking, dyspnea, wheezes, and chronic bronchitis were all reported to be significantly higher in the COPD–CAD group, compared with the non-COPD–CAD group (all OR >1, with 95% CI: beyond 1).

The OR in the COPD group was nearly twice that in the non-COPD group (OR: 1.94, 95% CI: 1.57–2.4). Moreover, a higher incidence of atrial fibrillation and a history of stroke were both observed in the group with comorbid COPD. Atrial fibrillation provided an OR of 1.64 (95% CI: 1.14–2.36), while a history of stroke generated an OR of 1.72 (95% CI: 1.35–2.18). See Table 2 for further details.

### 3.2 Impacts for COPD toward CAD

**Fig. 4.**
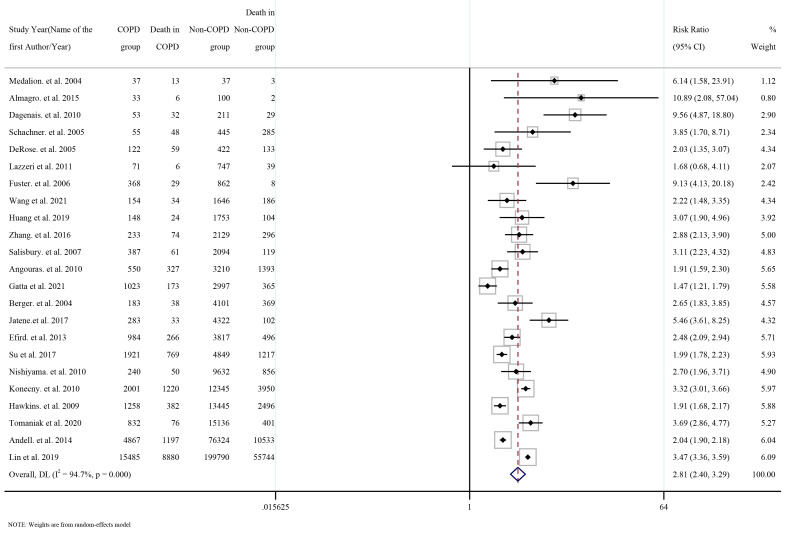
**Forest plot for risk of all-cause mortality according to COPD status**. COPD, chronic obstructive pulmonary disease.

A total of 23 studies reported all-cause mortality, 7 studies focused on cardiac death, 9 on myocardial infarction, and 6 focused on revascularization and stroke. Pooled all-cause mortality in the COPD group was triple that in the non-COPD group (risk ratio [RR] = 2.81, 95% CI: 2.40–3.29), see Fig. 4 and **Supplementary Table 6**.

Pooled cardiac death, myocardial infarction, stroke, heart failure, and respiratory failure were significantly higher in the COPD group than in the non-COPD group (all RR >1). However, pooled revascularization was lower in the COPD group than in the non-COPD group (RR = 0.86, 95% CI: 0.75–0.97), see **Supplementary Figs. 4–9**. Publication bias for different outcomes was also assessed using Egger’s test (**Supplementary Fig. 10**) and described below.

Sensitivity analyses, using the leave-one-out method, were conducted to determine whether any single study impacted the overall pooled RR (**Supplementary Fig. 11**). There was no obvious impact of a single study on all-cause death, cardiac death, myocardial infarction, stroke, heart failure, and respiratory failure. However, after excluding the study by Lin *et al*. [12] from the pooled RR for revascularization, the pooled RR increased, although, ultimately, remained lower than the null hypothesis, i.e., one (**Supplementary Fig. 11**).

A total of two studies directly compared the outcome of COPD–CAD patients who underwent CABG vs. PCI therapy. These two studies were both large, high-quality multicenter randomized clinical trials [8, 9]. However, they were also both post hoc, non-prespecified explorations. Therefore, we pooled the outcomes of these two studies, to compare the impact of the revascularization method on the COPD–CAD patients. As shown in **Supplementary Fig. 12**, an obvious reduction in revascularizations was observed after therapy (OR: 0.43, 95% CI: 0.20–0.94), along with a reduction in myocardial infarction (OR: 0.62, 95% CI: 0.18–2.11). However, no obvious benefit was observed from PCI for all-cause mortality (OR: 0.97, 95% CI: 0.54–1.74). The risk of stroke after revascularization increased in the CABG group (OR: 2.00, with 95% CI: 0.50–7.94).

Further subgroup analysis was conducted to investigate the differences in the RR between the PFT and ICD codes/self-reported method groups, and the CABG vs. PCI groups (**Supplementary Figs. 13,14**). Slightly higher mortality was observed in the PFT group compared with the ICD-codes/self-reported method group, although this was not considered significant (3.08 vs. 2.94, *p* value for subgroup differences = 0.833) (**Supplementary Fig. 14**). We also found no significant decrease in mortality in the CABG group compared to the PCI group (2.97 vs. 3.43, *p* for subgroup difference = 0.427) (**Supplementary Fig. 13**).

Studies reporting on revascularization in COPD–CAD patients were systematically reviewed. A total of seven studies reported the PCI rate for COPD–CAD patients. The pooled OR of the prescription rate in the COPD group was 0.68, with 95% CI: 0.56 to 0.83 when compared with the non-COPD group. A total of six studies reported the CABG rate for COPD–CAD patients and also indicated a reduced prescription rate for CABG in the COPD group, with a pooled OR equal to 0.93 and 95% CI: 0.75 to 1.15 (**Supplementary Fig. 15**). No significant difference was observed in strictly corrected COPD group according to GOLD criteria when compared with not strictly corrected COPD group (**Supplementary Fig. 16**).

### 3.3 Bias Assessment

Biases associated with the prevalence and outcomes were assessed separately, including publication bias and quality assessment. Prevalence-based publication bias was assessed using Begg’s and Egger’s tests; both at *p* > 0.05 (**Supplementary Fig. 2**). Publication bias for different outcomes was also assessed using Egger’s test (**Supplementary Fig. 10**). The main publication bias appeared to relate to the cardiac death and revascularization studies (cardiac death: Egger’s *p* = 0.027; revascularization: Egger’s *p* = 0.026). The risk of bias for prevalence and outcomes was independently assessed according to the methods described (**Supplementary Tables 7,8**).

## 4. Discussion

This systematic review and meta-analysis was designed to investigate global prevalence, comorbidities, and outcomes related to CAD patients with COPD. Additionally, we compared methods of revascularization and the outcomes for participants with COPD. We found a relatively high prevalence of COPD in CAD patients, which was higher than the previous estimate of 6% for the US adult population, provided in 2020 [74]. COPD-positive patients are more likely to be smokers, and hypertensive, with diabetes mellitus and atrial fibrillation, in addition to suffering from strokes. This supports the notion that there is a close relationship between COPD in CAD patients and other comorbidities. Additionally, we found that CAD patients with COPD are at high risk of all-cause death, cardiac death, myocardial infarction, stroke, heart failure, and respiratory failure. Further comparisons of CABG and PCI indicated that CABG may reduce the need for revascularization but that it did not lower the risk of death.

The prevalence of COPD in CAD patients is high, although there are also variations across different regions of the world. The highest rate of COPD in CAD is reported in North America, where the prevalence appears to be the same as the rate for COPD in atrial fibrillation [7]. One may assume that different diagnostic methods influence the prevalence, however, the diagnostics used for COPD are similar across North America, Europe, and Asia. Therefore, differences are more likely to be the result of culture, such as smoking and diet. Of course, there is a plethora of research on the link between diet and CAD, particularly around red meats, sugar, and salt [75, 76, 77], while the US, European nations, and Asia are distinct in terms of food cultures. Although, air pollution and other different epigenetic mechanisms can also create susceptibilities, as demonstrated by evidence that epigenetic mechanisms are involved in the development of COPD [78]; however, this does not account for differences in our genetic makeup. This study was not designed to explore genetic differences and we were only able to gain some insights into countries and cultures.

For example, we found one study that reported the prevalence of COPD–CAD in Africa. This study by Yangui *et al*. (2021) [16] was conducted in Tunisia, although it cannot be taken as representative since 98.3% of the sample participants were men, which suggests there are other issues that need to be overcome. For example, the high prevalence of COPD in CAD patients, at least in some Arab cultures in northern Africa, may relate to shisha culture, pollution, perhaps dry air, and socioeconomics. Cortes-Ramirez *et al*. [79] studied environmental risk factors associated with respiratory diseases in the region and found a potential link with Saharan dust. However, there is a paucity of evidence around the prevalence of COPD in African nations, generally [80]. Therefore, we have identified several issues that need to be studied to support health policymakers in African nations, not only related to smoking but in relation to the many other potential environmental and cultural factors involved.

We found a higher rate of smoking among those with comorbid COPD–CAD compared with CAD patients, without COPD. There is also strong evidence around the relationships between hypertension, diabetes mellitus, and COPD, with the accepted reason for this being tobacco smoking [81, 82]. COPD has also been identified as an independent factor that is involved in the development of atrial fibrillation [83, 84], while there is a higher incidence of stroke in those with COPD. According to several published studies, COPD influences stroke outcomes in two distinct ways, through COPD-related systemic inflammation and oxidative stress [85]. In the present study, all patients with CAD had similar risk ratios, which means the incidence of stroke may be due to cerebral vascular dysfunction or platelet hyperactivity related to COPD-related pathophysiologic mechanisms. Although, again, this is an area that demands further research.

Subgroup analysis highlighted differences among the included diagnostic methods. When the prevalence differences were compared, we found that PFT was associated with a 21.3% prevalence, ICD code diagnosis with 14.6%, and self-reported had 8.8%. One can assume this is related to the sensitivity and specificity of the diagnostic methods; however, perhaps more importantly, this highlights a potentially large clinical iceberg of CAD patients with COPD. This undiagnosed, and therefore untreated population is of particular concern because of the related outcomes and because many of these people may also be prediabetic or currently self-managing type II diabetes symptoms. Researchers have suggested that as much as 70% of the COPD population are undiagnosed, meaning they may be self-medicating or attempting to manage symptoms without knowing the exact cause [86]. This presents a number of problems and would certainly appear to support calls for more opportunistic testing while clinicians are treating patients for CAD.

We compared CABG to PCI and found that CABG had a similar risk ratio for mortality in the COPD group. This appears to contradict other studies that reported a beneficial effect on mortality from CABG for CAD patients. This result can be understood pathophysiologically since the occurrence of COPD and CAD is associated with systemic inflammation, oxygen depletion, and oxidative stress, which influence numerous coronary vessels. This, in turn, increases the probability of revascularization; however, this evidence was only generated from two randomized clinical trials, with small COPD patient samples. This of course affects the generalizability of the findings and the two clinical trials also did not categorize the COPD diagnostics as from either the pulmonary function test or any other test. This creates questions around the designs of studies and research quality and again highlights the need for further well-designed, clinical trials.

The incidence of revascularization in those with comorbid COPD–CAD did not increase above that observed in patients with CAD alone. Some studies have reported an increased incidence of MACEs in COPD patients after revascularization, which is mainly driven by mortality and not as a result of revascularization [10, 11]. This may explain why outcomes for COPD are so unsure, especially when choosing MACEs as the primary endpoint. Since revascularization is a MACE for COPD, MACEs are not the most suitable primary endpoint. Interestingly, there remains a substantial amount of publication bias with regard to revascularization outcomes. In a recent study, that adopted a leave-one-out approach, Lin *et al*. [12] found that revascularization had a substantial impact on pooled risk ratios. However, when we excluded the study by Lin *et al*. [12] from our analysis, the pooled risk ratio remained less than 1. This suggests that the impact of COPD on the outcome of CAD patients is limited, and therefore, revascularization may not influence outcomes as originally thought.

Several limitations ought to be discussed before we provide recommendations. First, we should acknowledge diagnostic biases, which will have occurred through different diagnostic methods. We must also acknowledge that more than half of the participants affected by COPD had not been diagnosed, which suggests the estimated prevalence of CAD–COPD is actually higher. Second, even though our study included numerous studies there are still some high-quality studies that were not included due to our inclusion criteria. However, this does not detract from the scientific merit of this study [87, 88]. Third, even though the goal was to assess global prevalence, we were not able to gain insights into African nations, most of the Middle East, South America, India, Central Asia, Southeast Asia, and Australia. One might assume this is related to income, although this was based on the heatmap rather than it being scientifically determined. Fourth, heterogeneity and bias appear particularly high and there are a number of reasons for this that should be further explored. Thus, additional research using a longitudinal approach and multinational databases is required, although this will require cooperation and collaboration at the highest levels. Finally, there appears to be an issue around polypharmacy reporting for those with COPD–CAD. This may be occurring because researchers feel it is unnecessary to report these interactions or because of publication parameters. We hope this will change; however, more sophisticated research designs are required for health policy development.

## 5. Conclusions

The global prevalence of COPD–CAD appears generally high, although there are clear geographical differences. COPD diagnostic methods undoubtedly cause a proportion of the variations observed, however, there is clearly a clinical iceberg of COPD among CAD patients. CAD patients with COPD also appear to have multiple related comorbidities, which influence prognoses. Physicians should opportunistically test for COPD to ensure their patients are not self-medicating and adding complications. More direct comparisons of revascularization versus anti-inflammation therapies, and beta-blockers for COPD–CAD patients may also prove useful.

## Data Availability

Datasets generated and analyzed for this work are available in the main text.

## Supplementary Material

Supplementary material associated with this article can be found, in the online version, at https://doi.org/10.31083/j.rcm2501025.
