# Supplementary material for: Global Insights into Chronic Obstructive Pulmonary Disease and Coronary Artery Disease: A Systematic Review and Meta-Analysis of 6,400,000 Patients
Source: Rev Cardiovasc Med. 2024 Jan 15;25(1):25. doi: 10.31083/j.rcm2501025 (PMC11262403; doi:10.31083/j.rcm2501025)
Supplement: Supplementary file 1 [file 2153-8174-25-1-025-s1.docx]

**Contents of Supplementary Materials**

P1 Title Page

P2 - P4 Supplementary material 1. Search Strategy

P5 - P6 Supplementary material 2. Customized NOS Tools

P7 - P23 Supplementary material 3. Supplementary Figures and Tables

P7 Supplementary Figure Legends

P8 Supplementary Fig. 1 Pooled prevalence of COPD in CAD patients

P9 Supplementary Fig. 2 Publication bias of pooled prevalence of COPD in CAD patients

P10 Supplementary Fig. 3 Leave-one-out analysis of pooled prevalence of COPD in CAD patients

P11 Supplementary Fig. 4 Forest plot of cardiac deaths risk according to COPD status

P12 Supplementary Fig. 5 Forest plot of myocardial infarction risk according to COPD status

P13 Supplementary Fig. 6 Forest plot of revascularization risk according to COPD status

P14 Supplementary Fig. 7 Forest plot of stroke risk according to COPD status

P15 Supplementary Fig. 8 Publication bias of pooled risk ratio of outcomes according to COPD status

P16 Supplementary Fig. 9 Leave-one-out analysis of pooled risk ratio of outcomes

P17 Supplementary Fig. 10 Publication bias of pooled risk ratio of outcomes

P18 Supplementary Fig. 11 Leave-one-out analysis of pooled risk ratio of outcomes

P19 Supplementary Fig. 12 Forest plot of revascularization methods according to COPD status

P20 Supplementary Fig. 13 Forest plot for all-cause mortality risk with revascularization subtype

P21 Supplementary Fig. 14 Forest plot of all-cause mortality risk with COPD diagnostic method

P22 Supplementary Fig. 15 Forest plot of outcomes according to revascularization method

P23 Supplementary Fig. 16 Prevalence of COPD in CAD in Strictly Correct Group

P24 Supplementary Table 1 Research characteristics of Enrolled Studies

P28 Supplementary Table 2 Sensitivity analysis of pooled prevalence according to analytical methods

P28 Supplementary Table 3 Sensitivity analysis for pooled prevalence according to exclusion of studies with sample size

P29 Supplementary Table 4 Univariate meta-regression according to prevalence of COPD in CAD

P29 Supplementary Table 5 Multivariate meta-regression according to prevalence of COPD in CAD

P30 Supplementary Table 6 Outcomes of CAD patients according to COPD status

P33 Supplementary Table 7 Quality assessment of COPD prevalence

P34 Supplementary Table 8 Quality assessment of COPD-CAD patient outcome

P35 Supplementary material 4. PRISMA Checklist

1

**Global Insights into Chronic Obstructive Pulmonary Disease and Coronary Artery Disease: A Systematic Review and Meta-analysis of 6,400,000 Patients**

**Supplemental material 1. Search Strategy**

***Pubmed：***

#1 Search: chronic obstructive pulmonary disease

#2 Search: Chronic Obstructive Lung Disease

#3 Search: COAD

#4 Search: COPD

#5 Search: Chronic Obstructive Airway Disease

#6 Search: Chronic Obstructive Pulmonary Disease

#7 Search: Chronic Airflow Obstructions

#8 Search: Chronic Airflow Obstruction

#9 Search: ((((((((chronic obstructive pulmonary disease) OR Chronic Obstructive Lung Disease) OR

COAD) OR COPD) OR Chronic Obstructive Airway Disease) OR Chronic Obstructive Pulmonary

Disease) OR Chronic Airflow Obstructions) OR Chronic Airflow Obstruction)

#10 Search: Coronary Artery Disease

#11 Search: Coronary Artery Diseases

#12 Search: Left Main Coronary Artery Disease

#13 Search: Left Main Disease

#14 Search: Left Main Diseases

#15 Search: Left Main Coronary Disease

#16 Search: Coronary Arteriosclerosis

#17 Search: Coronary Arterioscleroses

#18 Search: Coronary Atheroscleroses

2

#19 Search: Coronary Atherosclerosis

#20 Search: ((((((((((Coronary Artery Disease) OR Coronary Artery Diseases) OR Left Main Coronary

Artery Disease) OR Left Main Disease OR Left Main Diseases) OR Left Main Coronary Disease) OR

Coronary Arteriosclerosis) OR Coronary Arteriosclerosis) OR Coronary Arterioscleroses) OR

Coronary Atheroscleroses) OR Coronary Atherosclerosis)

#21 Search: ((((((((chronic obstructive pulmonary disease) OR Chronic Obstructive Lung Disease) OR

COAD) OR COPD) OR Chronic Obstructive Airway Disease) OR Chronic Obstructive Pulmonary

Disease) OR Chronic Airflow Obstructions) OR Chronic Airflow Obstruction) AND (((((((((( Coronary

Artery Disease ) OR Coronary Artery Diseases ) OR Left Main Coronary Artery Disease) OR Left

Main Disease OR Left Main Diseases) OR Left Main Coronary Disease) OR Coronary Arteriosclerosis)

OR Coronary Arteriosclerosis) OR Coronary Arterioscleroses) OR Coronary Atheroscleroses) OR

Coronary Atherosclerosis)

***Embase：***

#1 'chronic obstructive pulmonary disease'/ exp

#2 'chronic obstructive lung disease'/ exp

#3 'COPD'

#4 'chronic obstructive pulmonary disease'/ exp OR 'chronic obstructive lung disease'/ exp OR 'COPD'

#5 'coronary artery disease'/exp

#6 'coronary atherosclerosis'

#7 'coronary arteriosclerosis'

#8 'coronary artery disease'/exp OR 'coronary atherosclerosis' OR 'coronary arteriosclerosis'

3

#9 ('chronic obstructive pulmonary disease'/exp OR 'chronic obstructive lung disease'/exp OR 'COPD')

AND ('coronary artery disease'/exp OR 'coronary atherosclerosis' OR 'coronary arteriosclerosis')

***Web of Science：***

#1

TS

= (Chronic

obstructive

pulmonary

disease) OR TS = (Chronic obstructive

lung

disease) OR TS = (COPD)

#2 TS = (Coronary artery disease) OR TS = (Coronary artery diseases)

OR TS = (Coronary Atherosclerosis)

#3 #2 AND #1

***OpenGrey：***

(Chronic obstructive pulmonary disease OR Chronic obstructive lung disease OR COPD)

AND

(Coronary artery disease OR Coronary artery diseases OR Coronary Atherosclerosis）

***Chest：***

COPD and CAD

***European Heart Journal：***

COPD and CAD

4

**Supplemental material 2. Customized NOS Tools**

**Table 1 – Customized NOS for prevalence of COPD in CAD**

5

Selection

SAMPLE REPRESENTATIVENESS

- 1 point: Truly or somewhat representative of the average CAD in the community.
- 0 point: Not representative of the average CAD in the community (e.g. selected group of CAD patients)

Selection

SAMPLE SIZE

- 1 point: Sample size was greater than or equal to 1000 participants.
- 0 point: Sample size was less than 1000 participants.

Comparability

NON-RESPONDENTS

- 1 point: Comparability between respondent and non-respondent characteristics was established with a satisfactory response rate.
- 0 points: The comparability between respondents and non-respondents was unsatisfactory, the response rate was unsatisfactory, or there was no description of the response rate or the characteristics of the responders or non-responders.

Outcome

ASCERTAINMENT OF COPD

- 1 point: COPD was diagnosed through Pulmonary Function Test confirmation
- 0 point: COPD was diagnosed through medical history, self-reported methods, and ICD codes.

Outcome

QUALITY OF DESCRIPTIVE STATISTICS REPORTING

- 1 point: The study reported descriptive statistics to describe the population (e.g., age, sex, other comorbidities)
- 0 point: The study did not report descriptive statistics, or incompletely reported descriptive statistics

**Table 2 – Customized NOS for outcomes according to COPD status in CAD**

6

SELECTION

Sample representativeness:

- 1 point: Truly or somewhat representative of the average CAD in the community.
- 0 points: Not representative of the average CAD in the community (e.g. selected group of CAD patients)

COPD definition

- 1 point: COPD was diagnosed through Pulmonary Function Test confirmation.
- 0 point: COPD was diagnosed through medical history, self-reported methods, and ICD codes.

Selection of non-COPD patients

- 1 point: propensity-score matched, community controls, patients enrolled in the same setting
- 0 point: other type of controls/no description

Definition of non-COPD patients

- 1 point: non-COPD was confirmed through Pulmonary Function Test confirmation.
- 0 point: no description

COMPARABILITY (Max 2 points)

Comparability of COPD and non-COPD patients

- 1 point: COPD and non-COPD are sufficiently balanced for major risk scores (e.g. SYNTAX scores etc.)/imbalance not a major concern
- 1 point: COPD and non-COPD patients are sufficiently balanced also for other comorbidities/imbalance not a major concern

ENDPOINT

Ascertainment of endpoint

- 1 point: secure record (e.g. administrative databases, death certificates, etc.), or sufficiently reliable medical records
- 0 point: other/no description

Same method of ascertainment for COPD and non-COPD patients

- 1 point: yes
- 0 point: no

Non-response rate

- 1 point: same rate for both groups/differential rate was not a major concern
- 0 point: differential rate was a major concern

**Supplemental material 3. Supplementary Figures and Tables**

**Supplemental Figure Legends**

**Figure S1. Pooled prevalence of COPD in CAD patients**

COPD, chronic obstructive pulmonary disease; CAD, coronary artery disease; CI, confidence interval.

**Figure S2. Publication bias of pooled prevalence of COPD in CAD patients Figure S3. Leave-one-out analysis of pooled prevalence of COPD in CAD patients Figure S4. Forest plot of cardiac deaths risk according to COPD status**

**Figure S5. Forest plot of myocardial infarction risk according to COPD status Figure S6. Forest plot of revascularization risk according to COPD status Figure S7. Forest plot of stroke risk according to COPD status**

**Figure S8. Forest plot of heart failure risk according to COPD status Figure S9. Forest plot of respiratory failure risk according to COPD status**

**Figure S10. Publication bias of pooled risk ratio of outcomes according to COPD status Figure S11. Leave-one-out analysis of pooled risk ratio of outcomes according to COPD status**

**Figure S12. Forest plot of revascularization methods according to COPD status**

A. PCI prescription; B. CABG prescription. CABG, coronary artery bypass graft; PCI, percutaneous coronary intervention; CI, confidence interval; COPD, chronic obstructive pulmonary disease.

**Figure S13. Forest plot for all-cause mortality risk with revascularization subtype according to COPD status**

CAD, coronary artery disease; CABG, coronary artery bypass graft; PCI, percutaneous coronary intervention; CI, confidence interval.

**Figure S14. Forest plot of all-cause mortality risk with COPD diagnostic method subtype according to COPD status**

CAD, coronary artery disease; PFT, pulmonary function test; ICD/SRM, ICD-codes/Self-reported Methods; CI, confidence interval.

**Figure S15. Forest plot of outcomes according to revascularization method in COPD-CAD patients**

A. Outcome of all-cause death; B. Outcome of myocardial infarction; C. Outcome of revascularization;

D. Outcome of stroke. CABG, coronary artery bypass graft; PCI, percutaneous coronary intervention; CI, confidence interval; COPD, chronic obstructive pulmonary disease.

7


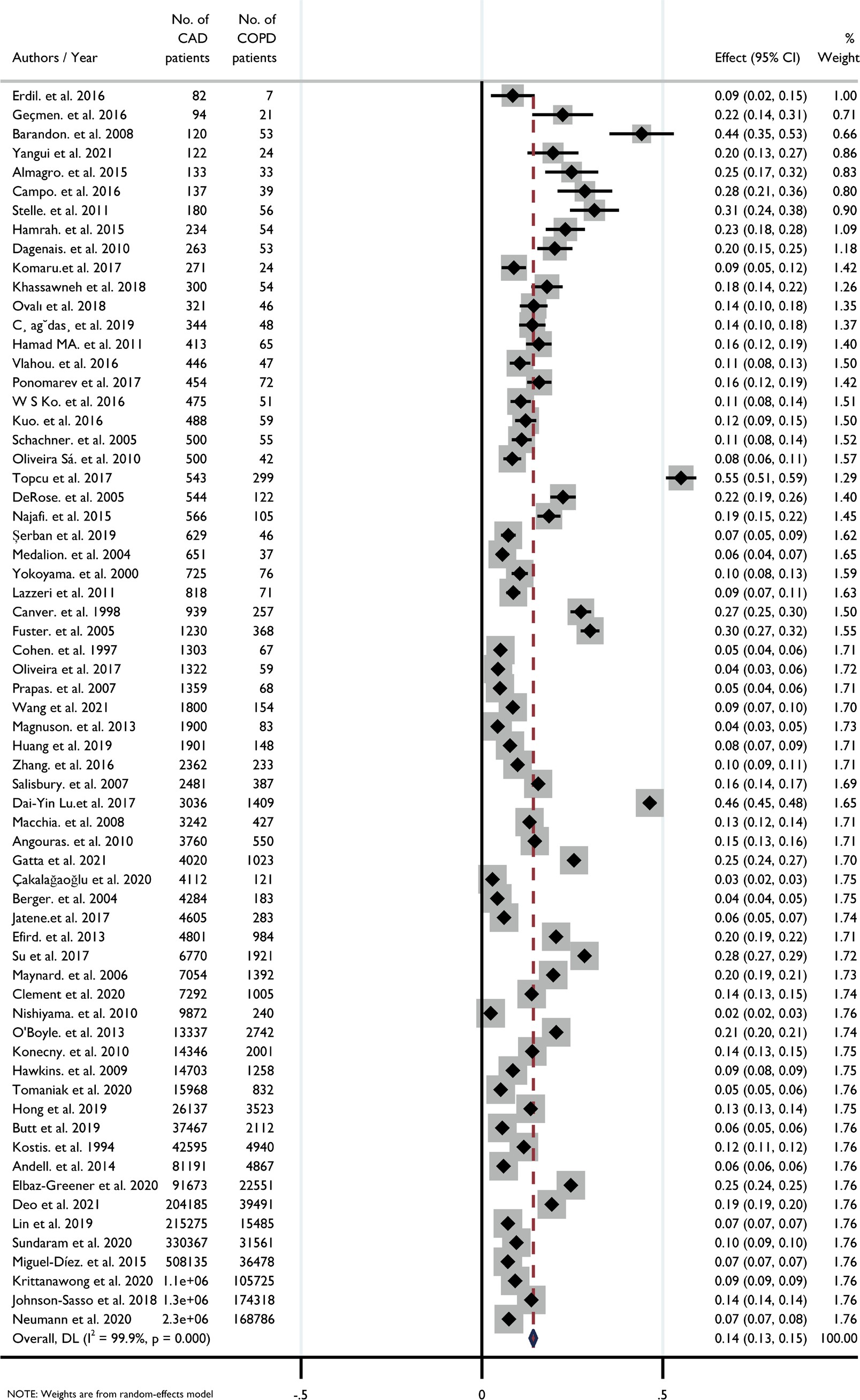


**Figure S1. Pooled prevalence of COPD in CAD patients**

COPD, chronic obstructive pulmonary disease; CAD, coronary artery disease; CI, confidence interval.

8


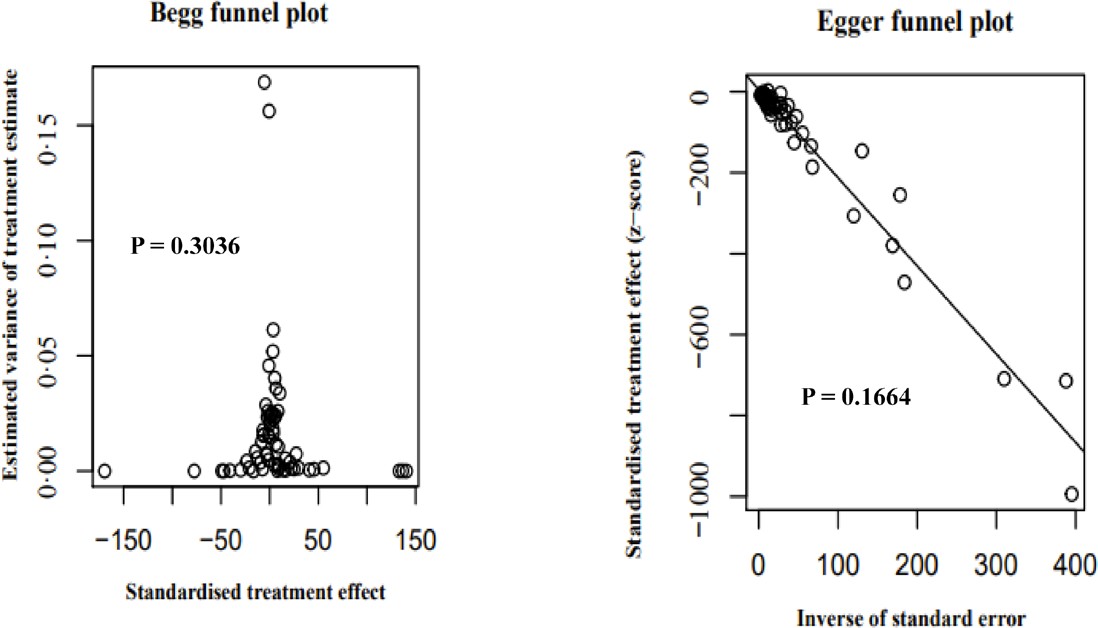


**Figure S2. Publication bias of pooled prevalence of COPD in CAD patients**

9


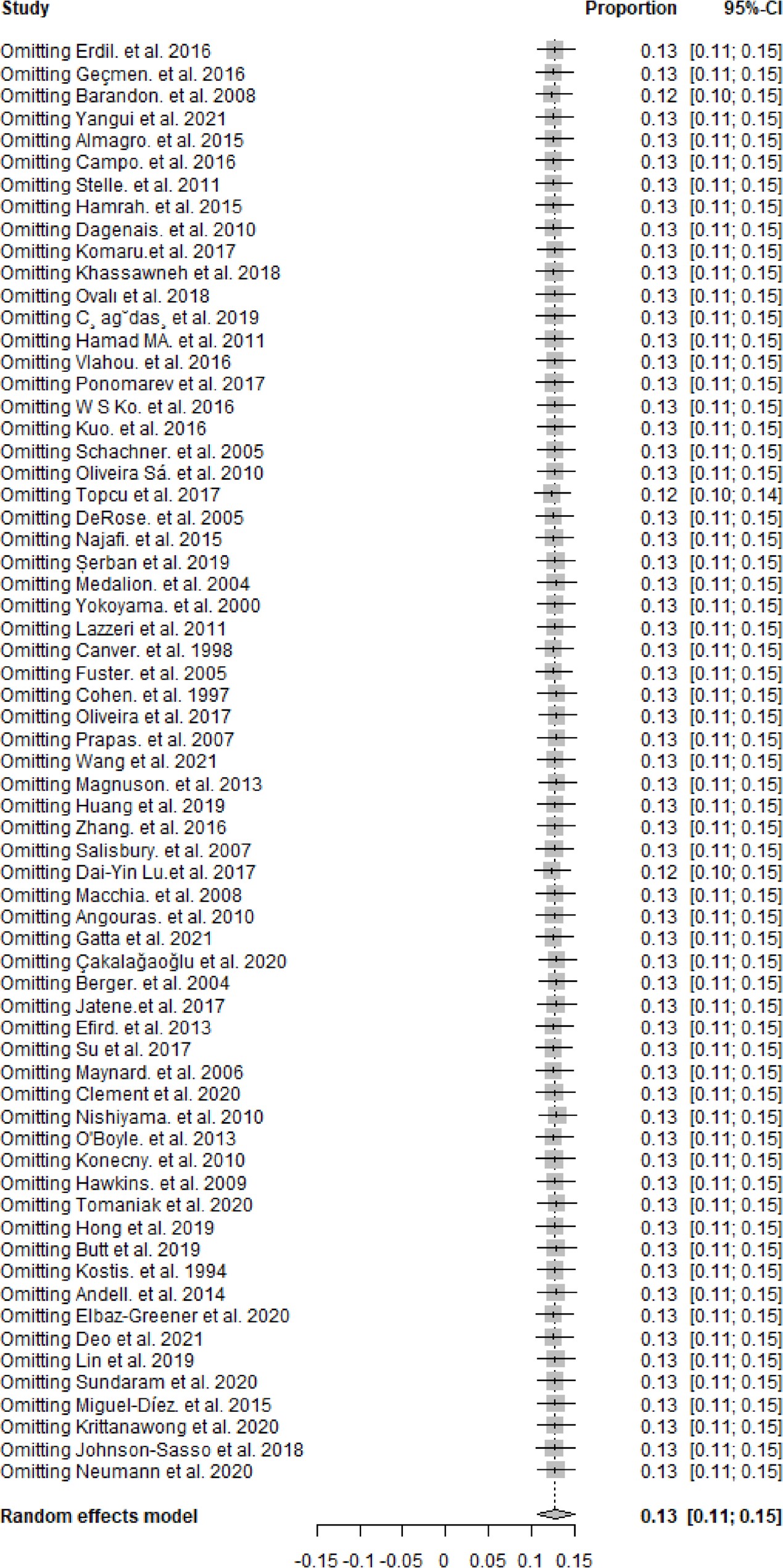


**Figure S3. Leave-one-out analysis of pooled prevalence of COPD in CAD patients**

10


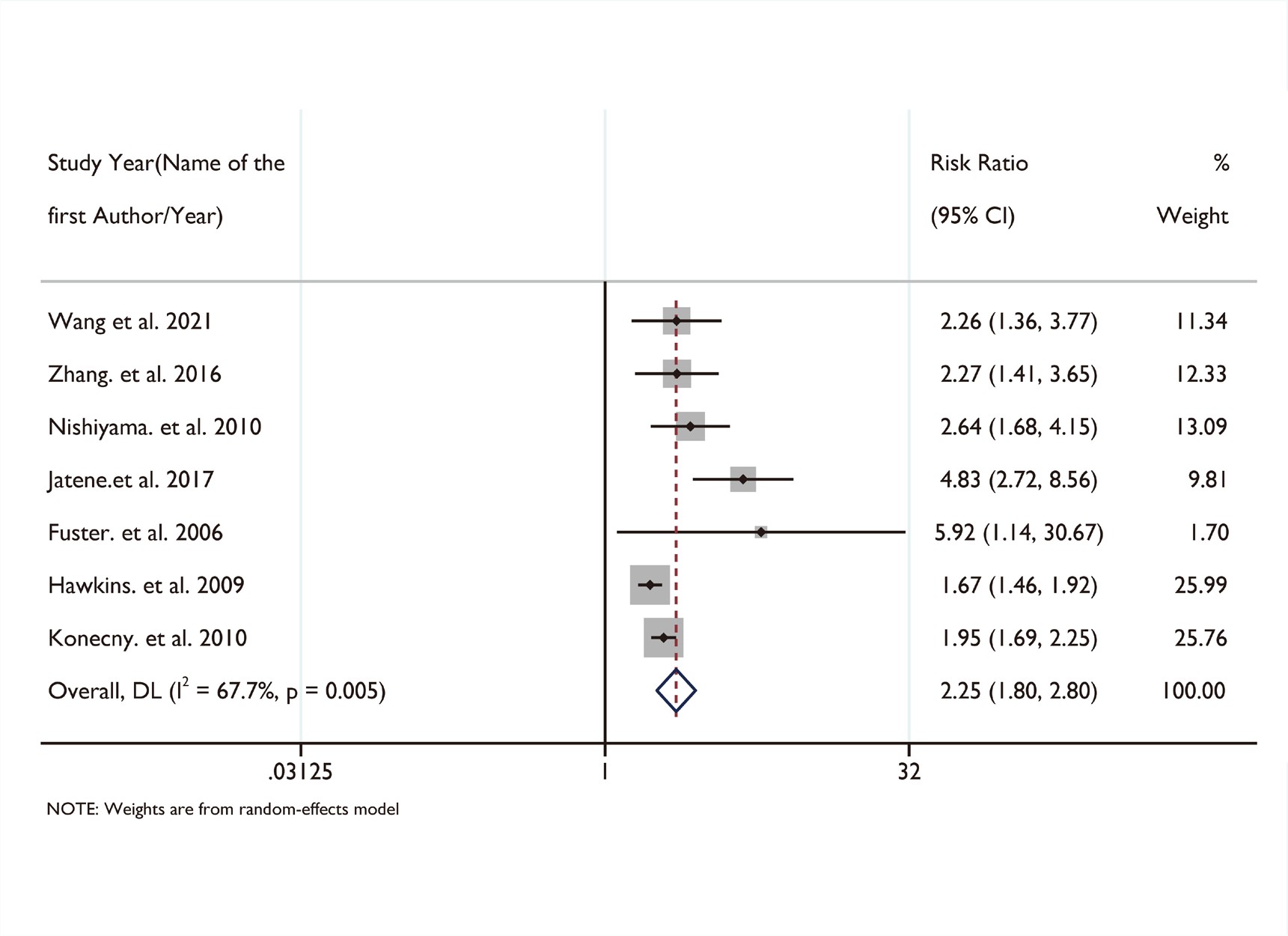


**Figure S4. Forest plot of cardiac deaths risk according to COPD status**

11


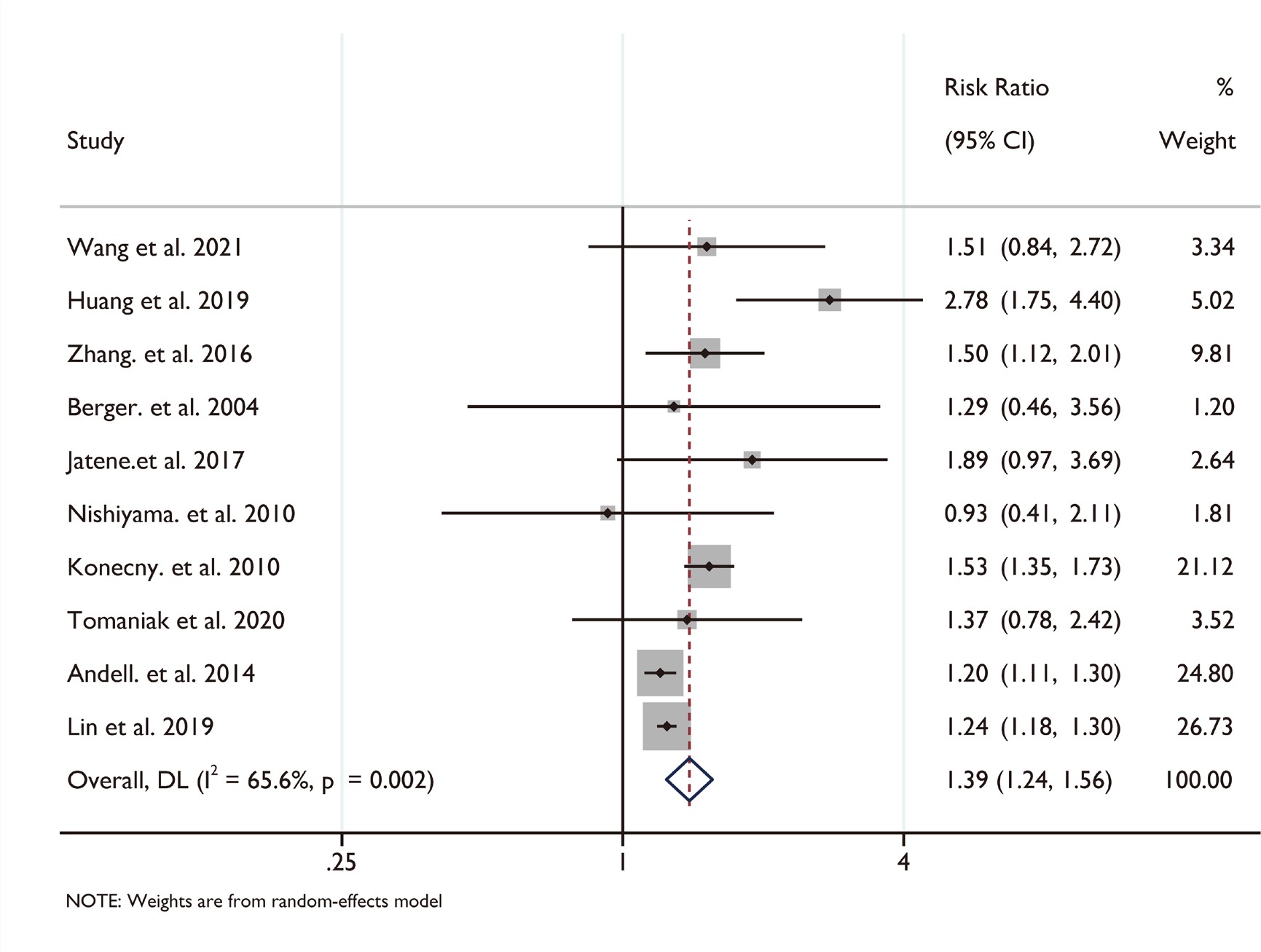


**Figure S5. Forest plot of myocardial infarction risk according to COPD status**

12


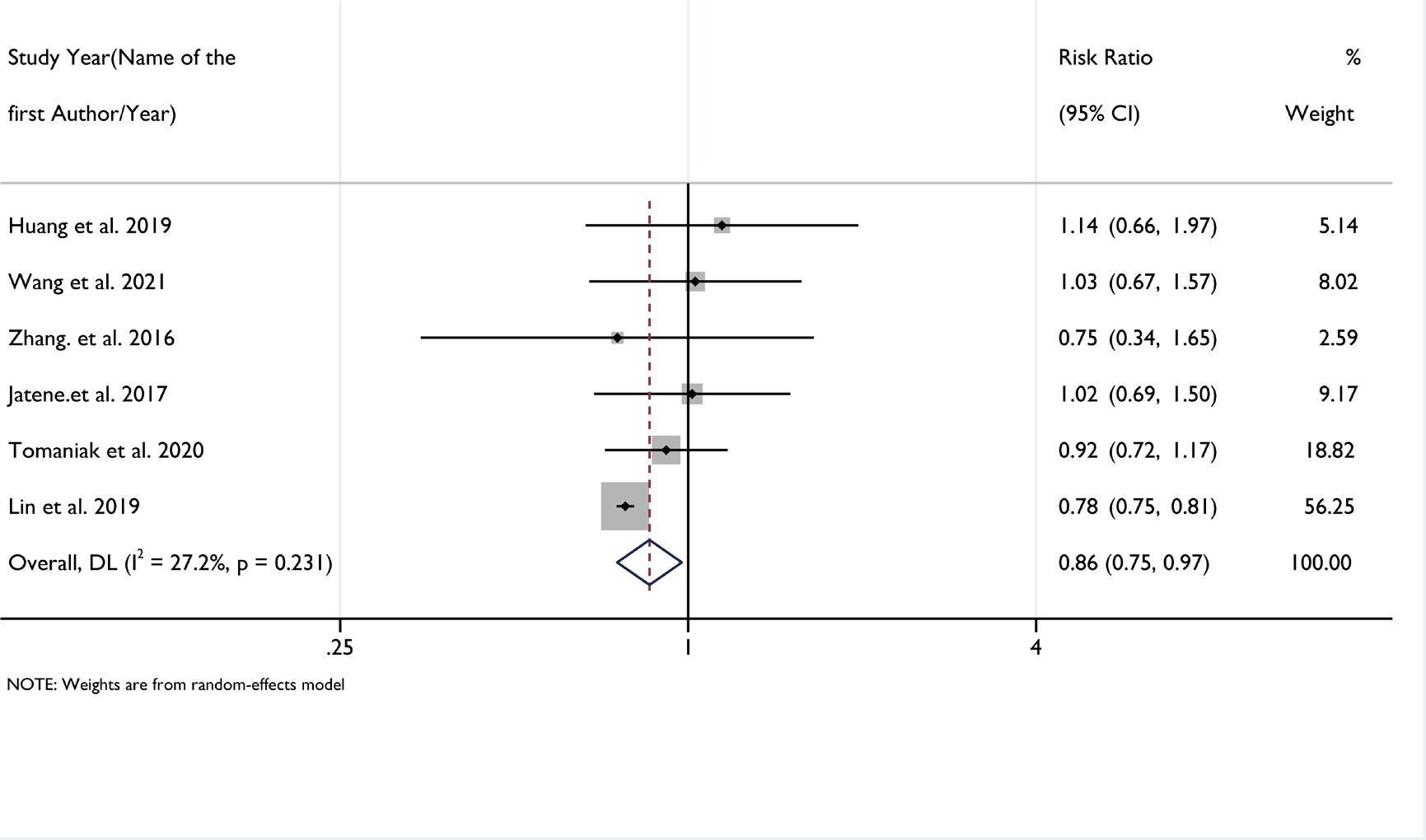


**Figure S6. Forest plot of revascularization risk according to COPD status**

13


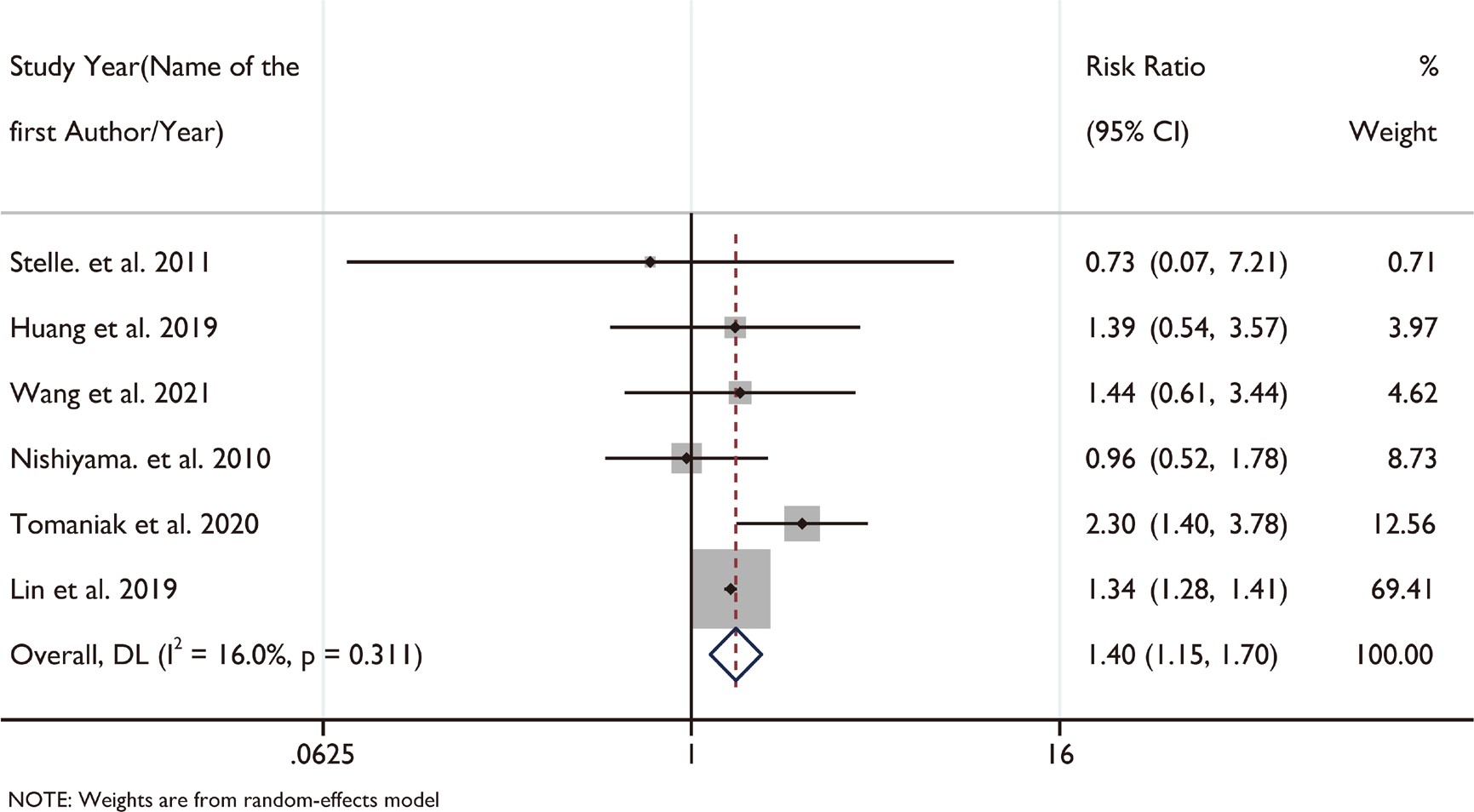


**Figure S7. Forest plot of stroke risk according to COPD status**

14


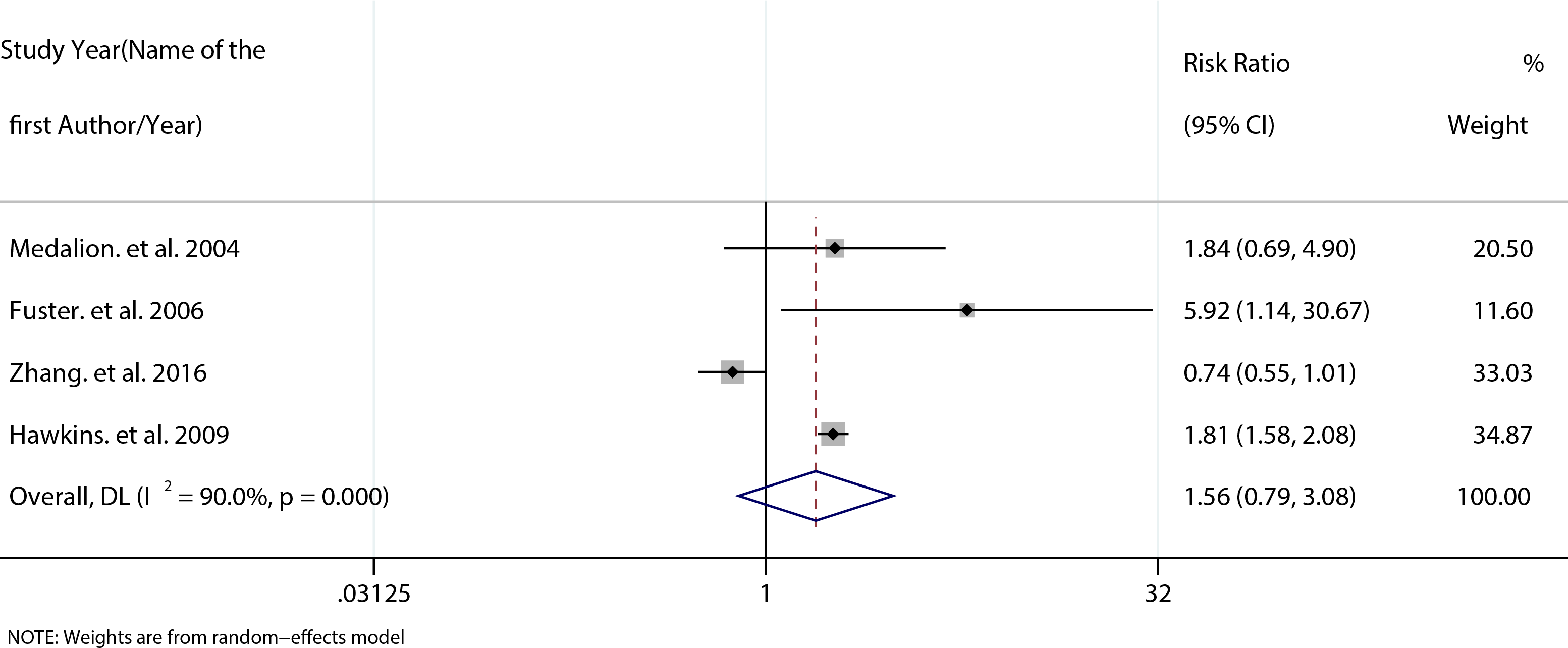


**Figure S8. Forest plot of heart failure risk according to COPD status**

15


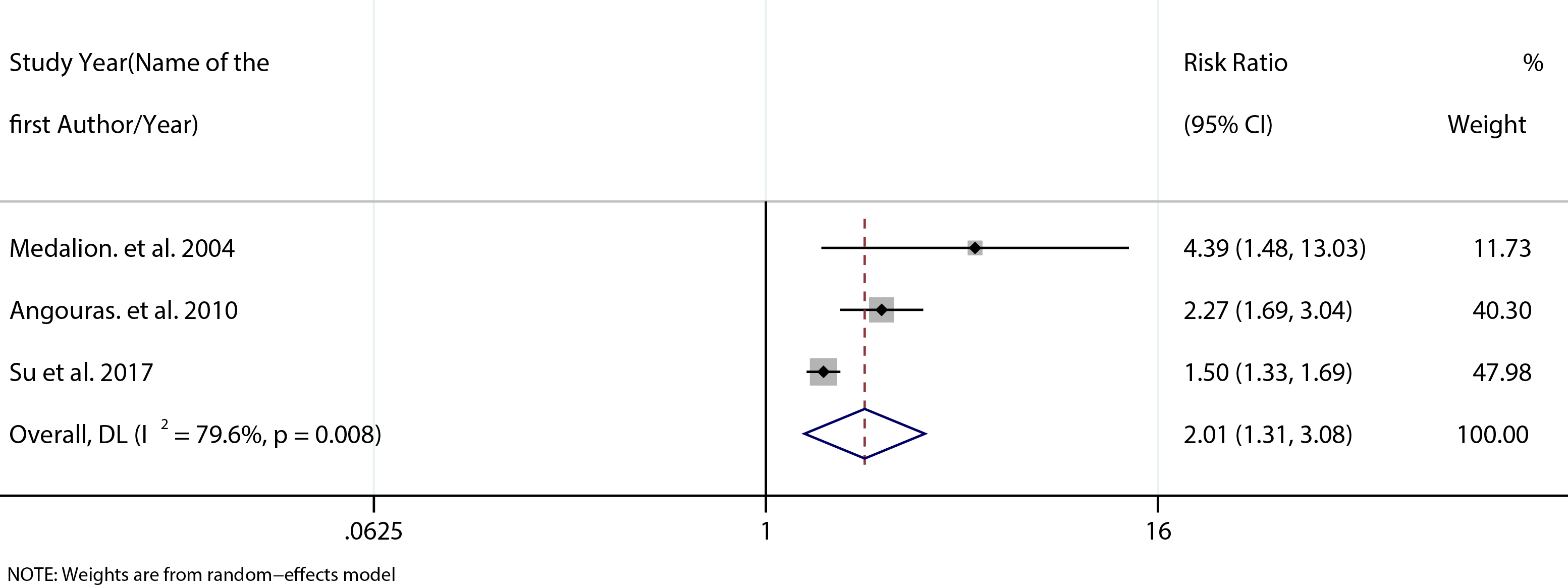


**Figure S9. Forest plot of respiratory failure risk according to COPD status**

16


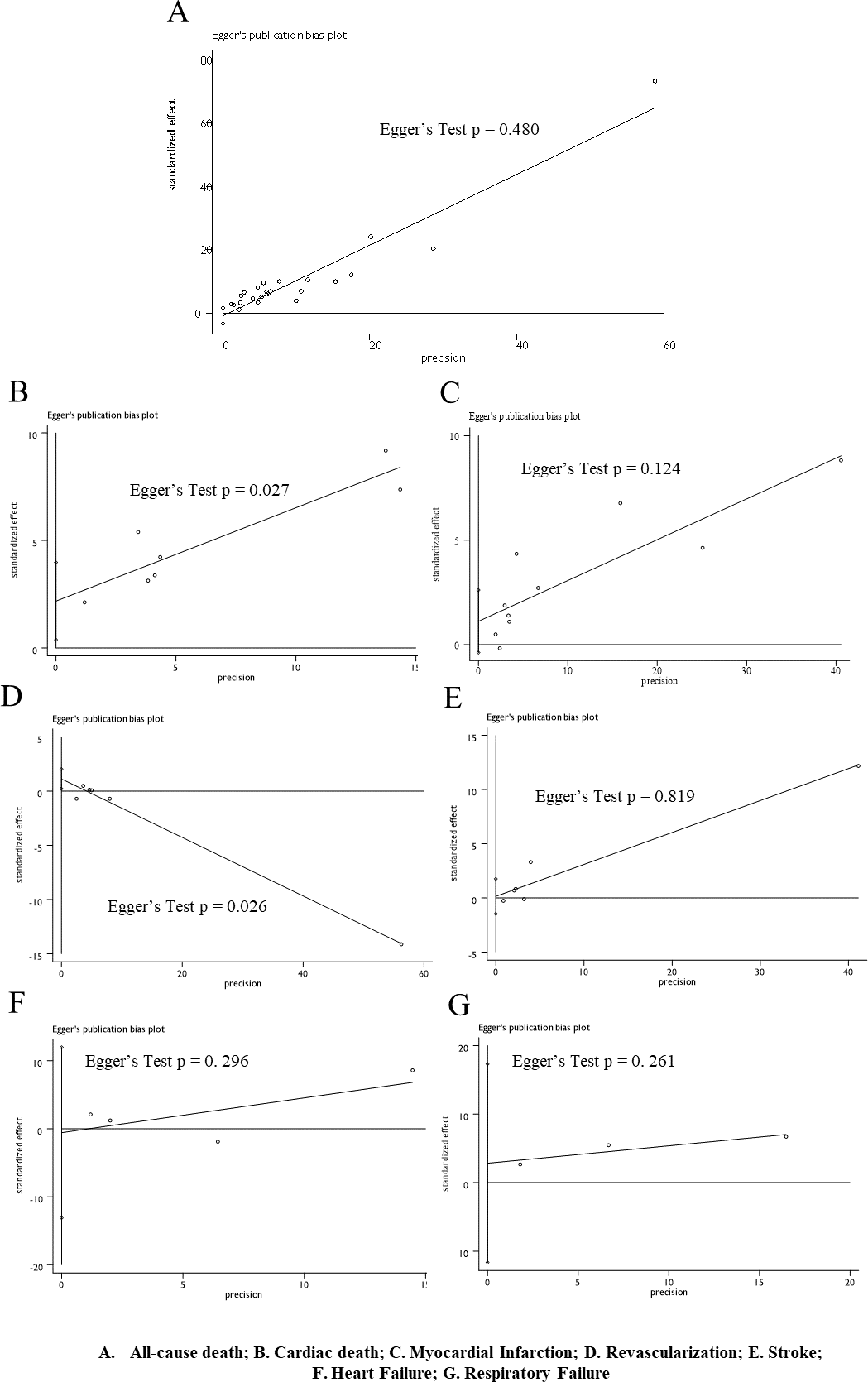


**Figure S10. Publication bias of pooled risk ratio of outcomes according to COPD status**

17


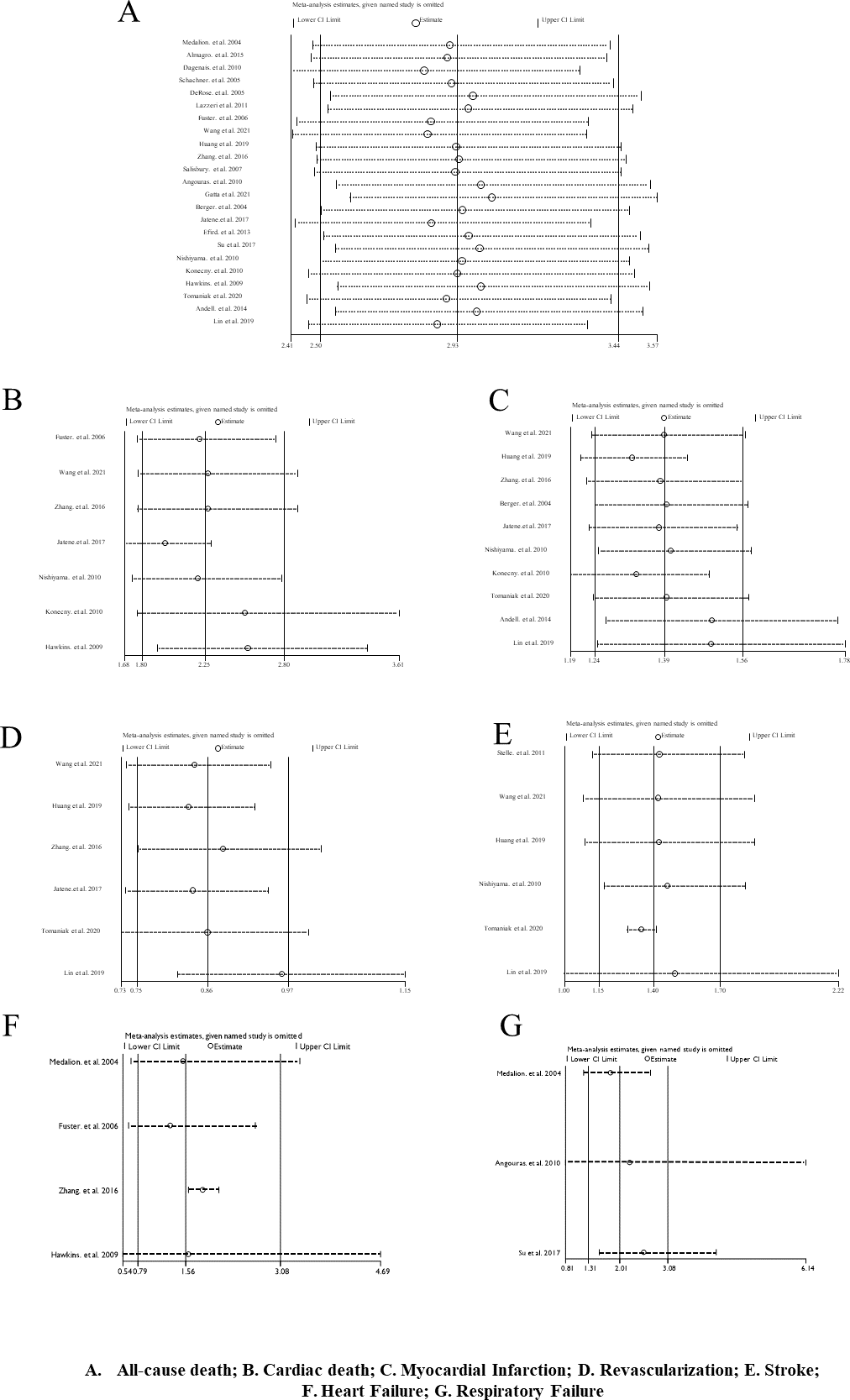


**Figure S11. Leave-one-out analysis of pooled risk ratio of outcomes according to COPD**

**status**

18


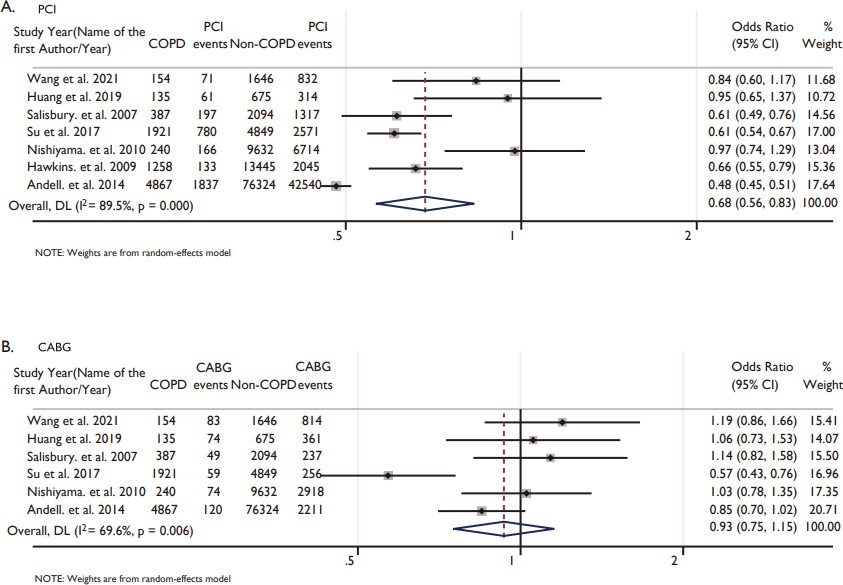


**Figure S12. Forest plot of revascularization methods according to COPD status**

A. PCI prescription; B. CABG prescription. CABG, coronary artery bypass graft; PCI, percutaneous coronary intervention; CI, confidence interval; COPD, chronic obstructive pulmonary disease.

19


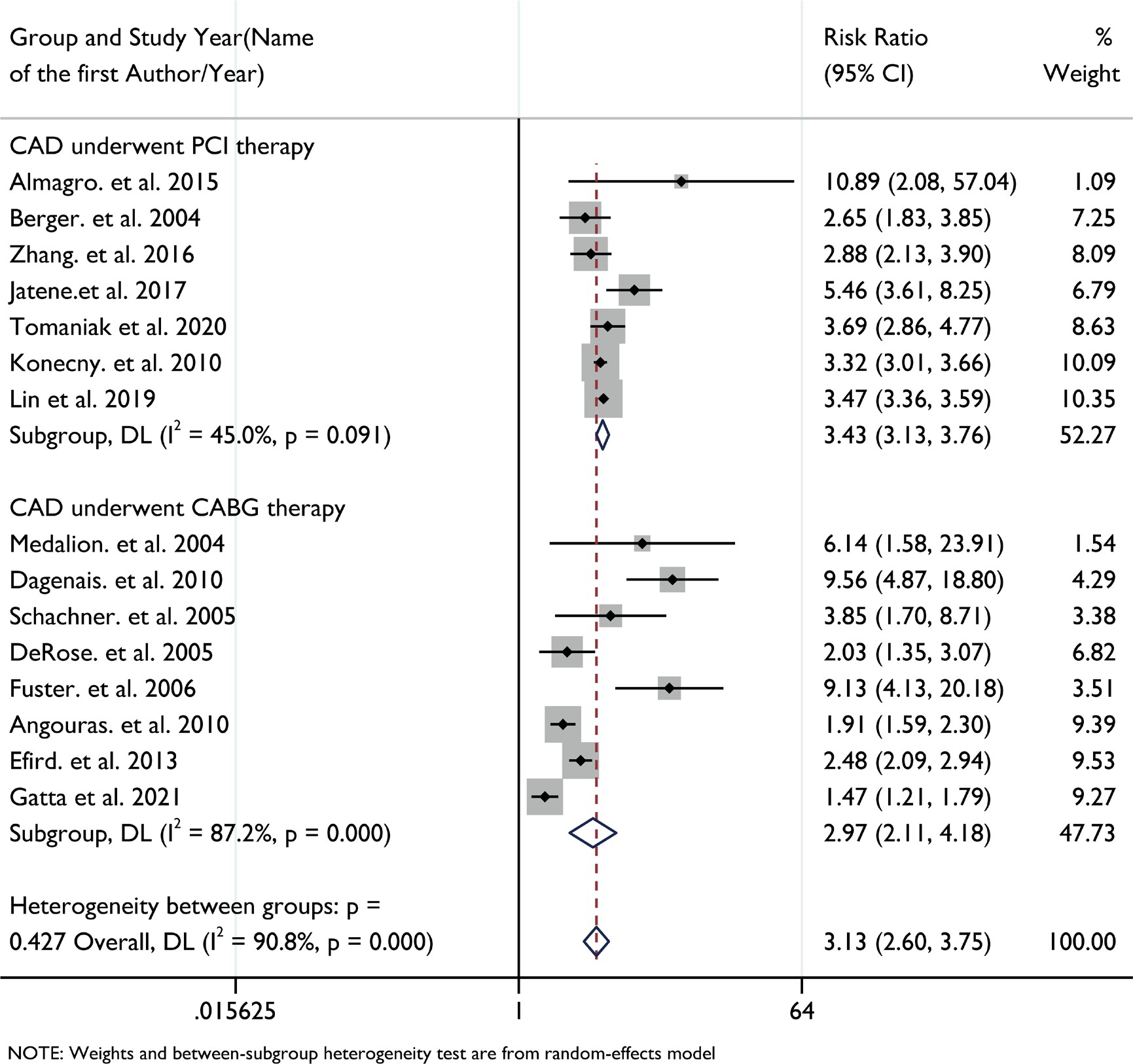


**Figure S13. Forest plot for all-cause mortality risk with revascularization subtype according to COPD status**

CAD, coronary artery disease; CABG, coronary artery bypass graft; PCI, percutaneous coronary

intervention; CI, confidence interval.

20


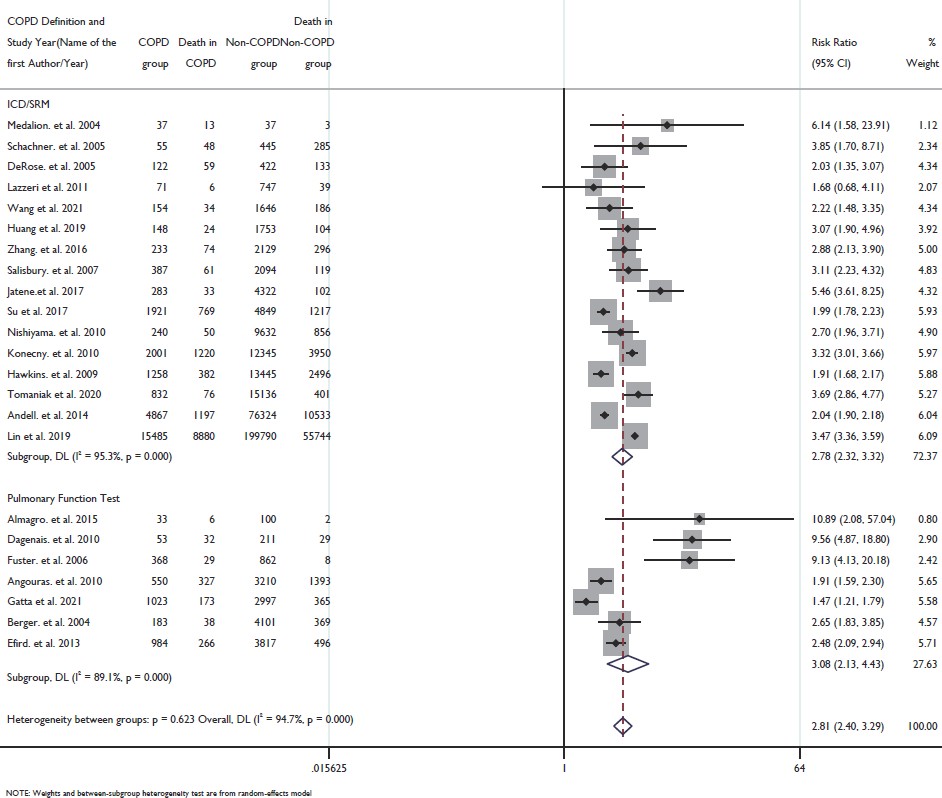


**Figure S14. Forest plot of all-cause mortality risk with COPD diagnostic method subtype according to COPD status**

CAD, coronary artery disease; PFT, pulmonary function test; ICD/SRM, ICD codes/Self-reported

Methods; CI, confidence interval.

21


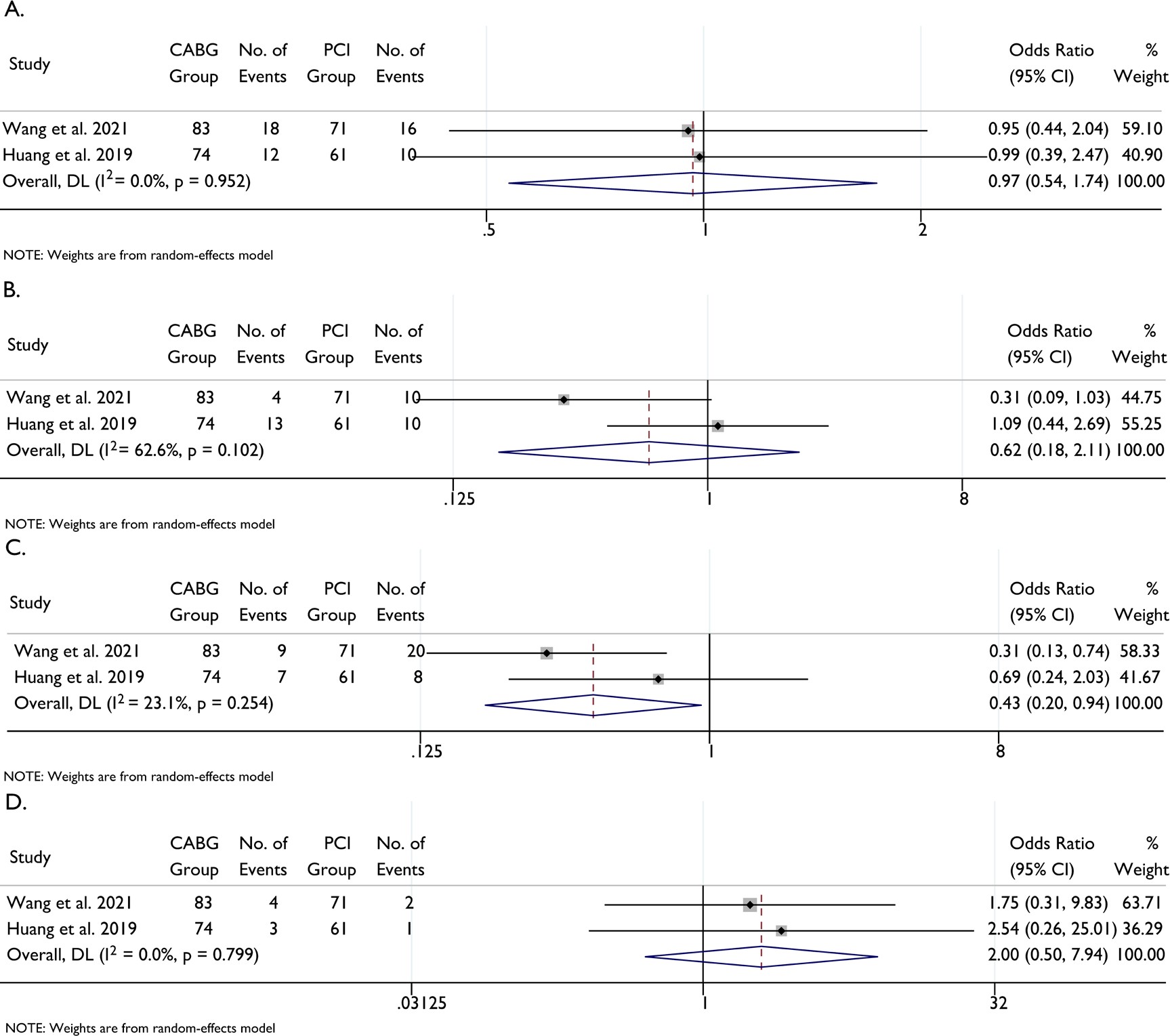


**Figure S15. Forest plot of outcomes according to revascularization method in COPD-CAD patients**

A. Outcome of all-cause death; B. Outcome of myocardial infarction; C. Outcome of revascularization;

D. Outcome of stroke. CABG, coronary artery bypass graft; PCI, percutaneous coronary intervention; CI, confidence interval; COPD, chronic obstructive pulmonary disease.

22


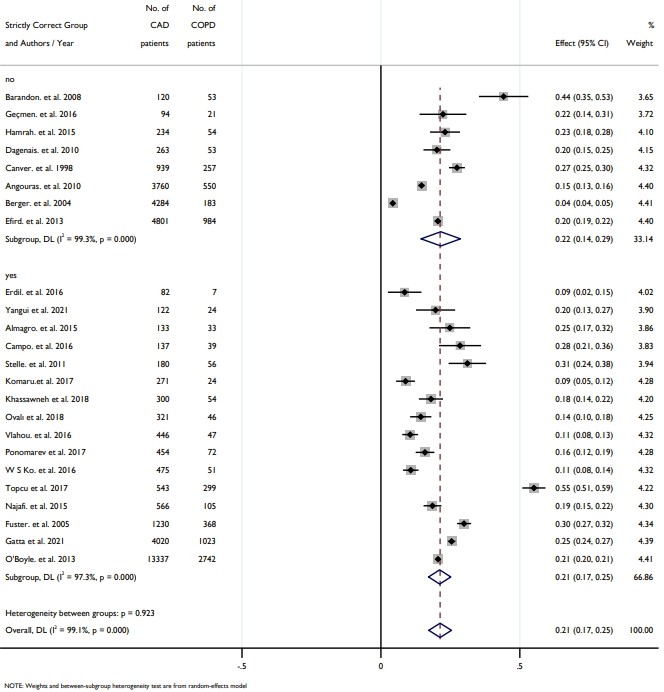


**Figure S16. Prevalence of COPD in CAD in Strictly Correct Group and**

**Non-strictly Correct Group according to GOLD Criteria**

**Table S1. Research characteristics of Enrolled Studies**

**Study Year (Name of**

**the first Author/Year)**

**Research held**

**Country or Region**

**Economic**

**Status**

**Diagnosed**

**Method**

**Area**

**Study Type**

**Patients Characteristics**

Upper middle income Upper middle

income

Observational Single Center Observational Single Center Observational Single Center Observational Single Center Observational Single Center Observational Single Center Observational Single Center Observational Single Center Observational Single Center Observational Single Center Observational Single Center Observational Single Center Observational

Single Center

Pulmonary Function Test

Pulmonary Function Test

Pulmonary Function Test

Pulmonary Function Test

Pulmonary Function Test

Pulmonary Function Test

Pulmonary Function Test

Pulmonary Function Test

Pulmonary Function Test

Pulmonary Function Test

Pulmonary Function Test

Pulmonary Function Test

Self-reported

Method

Erdil. et al. 201628

Turkey

Europe

CAD patients underwent CABG

Geçmen. et al. 201629

Turkey

Europe

CAD patients underwent CABG

Barandon. et al. 200830

France

Europe

High income

CAD patients underwent CABG

Upper middle

income

Yangui et al. 202131

Tunisia

Africa

CAD patients

Almagro. et al. 201532

Spain

Europe

High income

CAD patients underwent PCI

Campo. et al. 201633

Italy

Europe

High income

MI patients with smoking

United States Of

American

North

America

Stelle. et al. 201134

High income

CAD patients underwent CABG

Hamrah. et al. 201535

Japan

Asia

High income

CAD patients

North

America

CAD patients underwent CABG

over 70 years old

Dagenais. et al. 201036

Canada

High income

Komaru.et al. 201737

Japan

Asia

High income

CAD patients

Upper middle income Upper middle income Upper middle

income

Khassawneh et al. 201838

Jordan

Asia

CAD patients

Ovalı et al. 201839

Turkey

Europe

CAD patients underwent CABG

C¸ ag˘das¸ et al. 201940

Turkey

Europe

CAD patients underwent PCI

Observational

Single Center

CAD patients underwent CABG

with EF lower than 30%

Self-reported

Method

Hamad MA. et al. 201141

Netherlands

Europe

High income

Observational Single Center Observational Single Center Observational Single Center Adminstrative database Observational Single Center Observational

Single Center

Pulmonary Function Test

Pulmonary Function Test

Pulmonary Function

Test

Vlahou. et al. 201642

Greece

Europe

High income

CAD patients underwent CABG

Ponomarev et al. 201743

Russia

Europe

High income

CAD patients underwent CABG

Upper middle

income

W S Ko. et al. 201644

China

Asia

CAD patients underwent PCI

Taiwan region

(Province of China)

Kuo. et al. 201645

Asia

High income

MI patients

ICD codes

Self-reported Method

Self-reported

Method

Schachner. et al. 200546

Austria

Europe

High income

CAD patients underwent CABG

South

America

Upper middle

income

Oliveira Sá. et al. 201047

Brazil

CAD patients underwent CABG

24

Upper middle

income

Observational Single Center Observational Single Center Observational Single Center Observational Single Center Observational Single Center Observational Single Center Observational Single Center Observational Single Center Adminstrative database Observational Single Center Observational Single Center Observational Single Center Randomized Clinical Trial Randomized Clinical Trial Randomized Clinical Trial Observational Single Center Observational Multi Center Adminstrative database Adminstrative database Observational Single Center Observational Single Center Observational

Single Center

Pulmonary Function Test

Self-reported Method

Pulmonary Function Test

Self-reported Method

Self-reported Method

Self-reported Method

Self-reported Method

Pulmonary Function Test

Pulmonary Function Test

Self-reported Method

Self-reported Method

Self-reported Method

Self-reported

Method

Topcu et al. 201748

Turkey

Europe

CAD patients

United States Of

American

North

America

CAD patients underwent CABG

with EF<25%

DeRose. et al. 200549

High income

Upper middle income Upper middle

income

Najafi. et al. 201550

Iran

Asia

CAD patients underwent CABG

Șerban et al. 201951

Romania

Europe

MI patients

Medalion. et al. 200452

Israel

Asia

High income

CAD patients underwent CABG

United States Of

American

North

America

Yokoyama. et al. 200053

High income

CAD patients underwent CABG

Lazzeri et al. 201154

Italy

Europe

High income

MI patients underwent PCI

United States Of

American

North

America

Canver. et al. 199855

High income

CAD patients underwent CABG

Fuster. et al. 200556

Spain

Europe

High income

CAD patients underwent CABG

Cohen. et al. 199757

Israel

Asia

High income

CAD patients underwent CABG

South

America

Upper middle

income

Oliveira et al. 201758

Brazil

CAD patients underwent CABG

Prapas. et al. 200759

Greece

Europe

High income

CAD patients underwent CABG

CAD patients underwent

revascularization

Wang et al. 202111

Multiple Countries

N/A

N/A

Magnuson. et al. 201360

Multiple Countries

N/A

N/A

CAD patients with diabetes

ICD codes

CAD patients underwent

revascularization

Self-reported Method

Self-reported Method

Self-reported

Method

Huang et al. 201910

Multiple Countries

N/A

N/A

Upper middle

income

Zhang. et al. 201661

China

Asia

CAD patients underwent PCI

United States Of American Taiwan region

(Province of China)

North

America

Salisbury. et al. 200762

High income

MI patients

Dai-Yin Lu.et al. 201763

Asia

High income

CAD patients underwent CABG

ICD codes

Macchia. et al. 200864

Italy

Europe

High income

MI patients

ICD codes

Pulmonary Function Test

Pulmonary Function Test

Self-reported

Method

Angouras. et al. 201065

Greece

Europe

High income

CAD patients underwent CABG

Gatta et al. 202166

United Kingdom

Europe

High income

CAD patients underwent CABG

Upper middle

income

Çakalağaoğlu et al. 202067

Turkey

Europe

CAD patients underwent CABG

2*5*

United States Of

American

North

America

Observational Multi Center Randomized Clinical Trial Observational Single Center Adminstrative database Adminstrative database Adminstrative database Observational Multi Center Adminstrative database Observational Single Center Randomized Clinical Trial Randomized Clinical Trial Observational Single Center Adminstrative database Adminstrative database Observational Multi Center Adminstrative database Adminstrative database Adminstrative database Adminstrative database Adminstrative database Adminstrative database Adminstrative

database

Pulmonary Function Test

Self-reported Method

Pulmonary Function

Test

Berger. et al. 200468

High income

CAD patients underwent PCI

Jatene.et al. 201769

Multiple Countries

N/A

N/A

CAD patients underwent PCI

United States Of American Taiwan region

(Province of China) United States Of American

United States Of

American

North

America

Efird. et al. 201370

High income

CAD patients underwent CABG

Su et al. 201771

Asia

High income

MI patients

ICD codes

North America North

America

Maynard. et al. 200672

High income

MI patients

ICD codes

Clement et al. 202073

High income

CAD patients underwent CABG

ICD codes

CAD patients underwent

revascularization

Self-reported Method

Pulmonary Function

Test

Nishiyama. et al. 201074

Japan

Asia

High income

O'Boyle. et al. 201375

United Kingdom

Europe

High income

CAD patients underwent CABG

United States Of

American

North

America

Konecny. et al. 201076

High income

CAD patients underwent PCI

ICD codes

Self-reported Method

Self-reported Method

Self-reported

Method

Hawkins. et al. 200977

Multiple Countries

N/A

N/A

MI patients

Tomaniak et al. 202078

Multiple Countries

N/A

N/A

CAD patients underwent PCI

North

America

Hong et al. 201979

Canada

High income

CAD patients

Butt et al. 201980

Denmark

Europe

High income

CAD patients underwent CABG

ICD codes

United States Of

American

North

America

Kostis. et al. 199481

High income

MI patients

ICD codes

Andell. et al. 201482

Sweden

Europe

High income

MI patients

ICD codes

Elbaz-Greener et al.

202083

Israel

Asia

High income

MI patients underwent CABG

ICD codes

United States Of American Taiwan region

(Province of China)

North

America

Deo et al. 202184

High income

CAD patients underwent CABG

ICD codes

Lin et al. 201927

Asia

High income

CAD patients underwent PCI

ICD codes

Self-reported

Method

Sundaram et al. 202085

United Kingdom

Europe

High income

MI patients

CAD patients underwent

revascularization

Miguel-Díez. et al. 201586

Spain

Europe

High income

ICD codes

Krittanawong et al. 202087

Johnson-Sasso et al.

201888

United States Of American United States Of

American

North America North

America

High income

MI patients less than 55 years

ICD codes

High income

MI patients

ICD codes

26

Adminstrative

database

Neumann et al. 202089

Germany

Europe

High income

MI patients

ICD codes

COPD, Chronic Obstructive Pulmonary Disease; CAD, Coronary Artery Disease; MI, Mocardial Infarction; UA, Unable Angina; CABG, Coronary Artery Bypass Graft; PCI,

Percutaneous Coronary Intervention; ICD, International Classification of the Diseases; N/A, Not Applicable.

27

**Table S2. Sensitivity analysis of pooled prevalence according to analytical methods**

**Method**

**Prevalence**

**95%CI**

**Tau**

**Tau2**

**I2**

**Untransformed**

**proportions**

14.20%

13.3-15.1%

0.035

0.0012

99.90%

**IV, Logit**

**Transformation**

12.70%

10.7-15.0%

0.787

0.619

99.90%

**IV, FT**

13.90%

11.6-16.3%

0.14

0.0136

99.90%

IV = Inverse Variance; FT = Freeman-Tukey; CI, Confidence Interval.

**Table S3. Sensitivity analysis for pooled prevalence according to exclusion of studies with**

**sample size**

**No. of Studies Diagnosed**

**with PFT**

**No. of**

**Studies**

**Sample Size**

**Prevalence**

**95%CI**

**Tau**

**Tau2**

**I2**

**Overall Excluding sample sizes**

**<1000**

**Excluding sample sizes**

**<2500**

**Excluding sample sizes**

**<5000**

**Excluding sample sizes**

**<10000**

**Excluding sample sizes**

**<20000**

65

24

14.2%

13.3-15.1%

0.035

0.0012

99.9%

37

6

12.6%

11.5-13.8%

0.035

0.0012

100.0%

28

5

13.5%

12.2-14.8%

0.035

0.0012

100.0%

20

1

10.8-13.9%

0.035

0.0012

12.3%

100.0%

16

0

9.8-13.1%

0.033

0.0011

11.4%

100.0%

12

0

9.4-13.1%

0.033

0.0011

11.2%

100.0%

28

**Table S4. Univariate Meta-regression according to prevalence of COPD in CAD**

**Standard**

**error**

**Upper 95%**

**CI**

**P**

**value**

**Tau**

**square**

**R**

**square, %**

**Variables**

**Coefficient**

**Lower 95% CI**

**Univariate Meta-regression COPD Diagnosed Method** ICD codes

Self-reported Method Pulmonary Function Test **Economic Status**

High Income

Upper Middle Income N/A

**Study Type**

Adminstrative Database Observational Multi-center Observational Single-center Randomized Clinical Trial **Risk of Bias**

High Low **Area** Africa Asia Europe

North America South America N/A

**Age**

> 65 years

<= 65 years **Male Hypertension**

**Diabetes Mellitus Dyslipidemia Atrial Fibrillation History of Stroke**

**Smoker**

<0.0001

0.449

28.39

-

-0.4932

0.536

- 0.212

0.212

-

-0.9092

0.121

-

-0.0772

0.951

0.020

0.011

0.045

0.587

6.320

-

-0.1237

-0.8326

- 0.240

0.335

-

-0.5933

-1.4884

- 0.346

-0.1767

0.606

0.013

0.003

0.532

15.010

-

-1.0436

-0.0176

-0.8870

- 0.404

0.211

0.345

-

-1.8363

-0.4318

-1.5639

-

-0.2509

0.397

-0.2100

0.010

0.934

0.010

0.118

0.613

2.180

-

0.427

-

0.273

-

-0.1080

-

0.962

0.118

0.089

0.584

6.800

-

-0.5178

-0.4427

-0.2953

-1.3218

-1.2463

- 0.824

0.812

0.820

0.969

0.857

-

-2.1320

-2.0335

-1.9025

-3.2205

-2.9253

- 1.097

1.148

1.312

0.577

0.433

0.530

0.586

0.719

0.172

0.146

0.652

0.631

0.000

-

-0.1003

0.015

0.013

0.017

0.011

-0.008

0.038

0.006

- 0.222

0.011

0.007

0.008

0.007

0.026

0.041

0.006

-

-0.5358

-0.0061

-0.0007

0.001

-0.0032

-0.0589

-0.0425

-0.0061

- 0.335

0.037

0.027

0.034

0.025

0.043

0.118

0.018

0.652

0.160

0.063

0.038

0.132

0.765

0.358

0.336

0.624

0.594

0.528

0.556

0.858

0.669

0.657

1.780

4.660

5.760

4.040

0.000

0.000

0.000

COPD: Chronic Obstructive Pulmonary Intervention; CAD, Coronary Artery Disease; ICD, International Classfication

of the Diseases; CI, Confidence Interval; N/A, Not Applicable.

29

**Table S5. Multivariate Meta-regression according to prevalence of COPD in CAD**

**Standard**

**error**

**Upper 95%**

**CI**

**P**

**value**

**Tau**

**square**

**R**

**square, %**

**Variables**

**Coefficient**

**Lower 95% CI**

**Overall**

**COPD Diagnosed Method** Self-reported Method Pulmonary Function Test ICD codes

**Economic Status**

High Income

Upper Middle Income N/A

**Study Type**

Adminstrative Database Observational Multi-center Observational Single-center Randomized Clinical Trial **Diabetes Mellitus**

0.002

0.429

23.64

-0.1535

0.490

-

0.359

0.344

-

-0.8580

-0.1841

-

0.551

1.164

-

0.669

0.154

-

-0.2389

-0.5820

- 0.257

0.458

-

-0.7428

-1.4795

- 0.265

0.315

0.353

0.204

-

-0.9057

-0.0747

- 0.007

- 0.444

0.334

- 0.009

-

-1.7757

-0.7299

-

-0.0098

-

-0.0357

0.581

- 0.023

0.041

0.823

-

0.420

COPD: Chronic Obstructive Pulmonary Intervention; CAD, Coronary Artery Disease; ICD, International

Classfication of the Diseases; CI, Confidence Interval; N/A, Not Applicable.

30

**Table S6. Outcomes of CAD patients according to COPD status**

**COPD Group**

**Non-COPD Group**

**Study Year(Name of the first**

**Author/Year)**

**Total COP D**

**Partic**

**ipants**

**No. of Myocar dial Infarcti**

**on**

**Total Non- COPD**

**Particip**

**ants**

**No. of Myocar dial Infarctio**

**n**

**No. of MA**

**CE**

**No. of All- cause**

**death**

**No. of Cardi ac**

**death**

**No. of Revas culari**

**zation**

**No. of Stro**

**ke**

**No. of Respirat ory**

**Failure**

**No. of MA**

**CE**

**No. of All- cause**

**death**

**No. of Revascu larizatio**

**n**

**No. of Heart**

**Failure**

**Age, year**

**s**

**No. of Cardiac**

**death**

**No. of Strok**

**e**

**No. of Heart**

**Failure**

**No. of**

**Respirato ry Failure**

**Age,**

**years**

Medalion. et al. 2004

Almagro. et al. 2015

Dagenais. et al. 2010

Schachner. et al. 2005

DeRose. et al. 2005

Lazzeri et al. 2011 Fuster. et al. 2006 Wang et al. 2021 Huang et al. 2019 Zhang. et al. 2016 Salisbury. et al.

2007

37

63.5

N/A

13

N/A

N/A

N/A

N/A

27

17

37

64.5

N/A

3

N/A

N/A

N/A

N/A

22

6

33

67.5

N/A

6

N/A

N/A

N/A

N/A

N/A

N/A

100

61.6

N/A

2

N/A

N/A

N/A

N/A

N/A

N/A

53

N/A

N/A

32

N/A

N/A

N/A

N/A

N/A

N/A

211

N/A

N/A

29

N/A

N/A

N/A

N/A

N/A

N/A

55

N/A

N/A

48

N/A

N/A

N/A

N/A

N/A

N/A

445

N/A

N/A

285

N/A

N/A

N/A

N/A

N/A

N/A

122

N/A

N/A

59

N/A

N/A

N/A

N/A

N/A

N/A

422

N/A

N/A

133

N/A

N/A

N/A

N/A

N/A

N/A

71

368

154

148

233

74

64.9

66.9

67.2

66.4

N/A N/A 61

44

N/A

6

29

34

24

74

N/A 5

20

N/A 23

N/A N/A 14

26

74

N/A N/A 29

16

7

N/A N/A 6

5

N/A

N/A 5

N/A N/A

62

N/A N/A N/A N/A

N/A

747

862

1646

1753

2129

67

62.4

64.9

65.9

63.9

N/A N/A 500

234

N/A

39

8

186

104

296

N/A 2

102

N/A 98

N/A N/A 102

125

504

N/A N/A 303

168

84

N/A N/A 45

43

N/A

N/A 2

N/A

N/A 697

N/A N/A N/A N/A

N/A

387

64.5

N/A

61

N/A

N/A

N/A

N/A

N/A

N/A

2094

60.1

N/A

119

N/A

N/A

N/A

N/A

N/A

N/A

31

Angouras. et al. 2010

Gatta et al. 2021 Berger. et al. 2004 Jatene.et al. 2017 Efird. et al. 2013 Su et al. 2017 Nishiyama. et al. 2010

Konecny. et al. 2010

Hawkins. et al. 2009

Tomaniak et al. 2020

Andell. et al. 2014

550

65.2

N/A

327

N/A

N/A

N/A

N/A

N/A

68

3210

64

N/A

1393

N/A

N/A

N/A

N/A

N/A

188

1023

183

283

984

1921

68.4

66.1

67.8

63.8

77

N/A 5

43

N/A N/A

173

38

33

266

769

N/A N/A 16

N/A

N/A

N/A 4

10

N/A N/A

N/A N/A 30

N/A

N/A

N/A N/A N/A N/A

N/A

N/A N/A N/A N/A

N/A

N/A N/A N/A N/A

573

2997

4101

4322

3817

4849

66.4

63.3

63

64

67

N/A 123

351

N/A N/A

365

369

102

496

1217

N/A N/A 53

N/A

N/A

N/A 70

82

N/A N/A

N/A N/A 452

N/A

N/A

N/A N/A N/A N/A

N/A

N/A N/A N/A N/A

N/A

N/A N/A N/A N/A

1069

240

N/A

7

50

22

6

N/A

11

N/A

N/A

9632

N/A

235

856

354

259

N/A

457

N/A

N/A

2001

69.9

N/A

1220

280

378

N/A

N/A

N/A

N/A

12345

66

N/A

3950

951

1629

N/A

N/A

N/A

N/A

1258

68.1

N/A

382

307

N/A

N/A

N/A

317

N/A

13445

64.5

N/A

2496

2177

N/A

N/A

N/A

2107

N/A

832

67.9

87

76

N/A

13

74

18

N/A

N/A

15136

64.4

566

401

N/A

173

1458

144

N/A

N/A

4867

75

N/A 1187

7

1197

N/A

808

N/A

N/A

N/A

N/A

76324

70

N/A 1205

31

10533

N/A

10838

N/A

N/A

N/A

N/A

Lin et al. 2019

15485

74

8880

N/A

2072

5019

2155

N/A

N/A

199790

64.7

55744

N/A

22086

76189

21445

N/A

N/A

32

**Table S7. Quality assessment of COPD prevalence**

**Study Year (Name of the first Author/Year)**

**Selection**

**Comparability**

**Outcome**

**Total**

Kostis. et al. 1994 Cohen. et al. 1997 Canver. et al. 1998 Yokoyama. et al. 2000 Berger. et al. 2004 Medalion. et al. 2004 DeRose. et al. 2005 Schachner. et al. 2005 Fuster. et al. 2006 Maynard. et al. 2006 Prapas. et al. 2007 Salisbury. et al. 2007 Barandon. et al. 2008 Macchia. et al. 2008 Hawkins. et al. 2009 Angouras. et al. 2010 Oliveira Sá. et al. 2010 Dagenais. et al. 2010 Konecny. et al. 2010 Nishiyama. et al. 2010 Hamad MA. et al. 2011 Stelle. et al. 2011 Lazzeri et al. 2011 Efird. et al. 2013 Magnuson. et al. 2013 O'Boyle. et al. 2013 Andell. et al. 2014 Almagro. et al. 2015 Miguel-Díez. et al. 2015 Hamrah. et al. 2015 Najafi. et al. 2015 Campo. et al. 2016 Erdil. et al. 2016 Geçmen. et al. 2016

W S Ko. et al. 2016 Kuo. et al. 2016 Vlahou. et al. 2016 Zhang. et al. 2016 Jatene.et al. 2017

Komaru.et al. 2017 Dai-Yin Lu.et al. 2017 Oliveira et al. 2017 Ponomarev et al. 2017 Su et al. 2017

Topcu et al. 2017 Johnson-Sasso et al. 2018 Khassawneh et al. 2018 Ovalı et al. 2018

Butt et al. 2019

C¸ ag˘das¸ et al. 2019 Hong et al. 2019 Huang et al. 2019

Lin et al. 2019

Șerban et al. 2019 Çakalağaoğlu et al. 2020 Clement et al. 2020

Elbaz-Greener et al. 2020 Krittanawong et al. 2020 Neumann et al. 2020 Sundaram et al. 2020 Tomaniak et al. 2020 Deo et al. 2021

Gatta et al. 2021

Wang et al. 2021 Yangui et al. 2021

2

2

1

1

2

1

1

1

2

2

2

2

1

2

2

2

1

1

2

2

1

1

1

2

2

2

2

1

2

1

1

1

1

1

1

1

1

2

2

1

2

2

1

2

1

2

1

1

2

1

2

2

2

1

2

2

2

1

2

2

2

2

2

2

1

1

1

1

1

1

1

1

1

1

1

1

1

1

1

1

1

1

1

1

1

1

1

1

1

1

1

1

1

1

1

1

1

1

1

1

1

1

1

1

1

1

1

1

1

1

1

1

1

1

1

1

1

1

1

1

1

1

1

1

1

1

1

1

1

1

1

0

2

1

2

1

1

1

2

1

1

1

2

1

1

2

1

2

1

1

1

2

1

2

2

1

1

2

1

2

2

2

2

2

2

1

2

1

1

2

1

1

2

1

2

1

2

1

1

1

1

1

1

1

1

1

1

1

1

1

1

1

2

1

2

4

3*

4

3*

5

3*

3*

3*

5

4

4

4

4

4

4

5

3*

4

4

4

3*

4

3*

5

5

4

4

4

4

4

4

4

4

4

4

3*

4

4

4

4

4

4

4

4

4

4

4

3*

4

3*

4

4

4

3*

4

4

4

3*

4

4

4

4

5

4

4

*: Study at high risk of bias, with total score <= 3.

COPD, Chronic Obstructive Pulmonary Disease; NOS, Newcastle–Ottawa Scale.

33

**Table S8. Quality assessment of COPD-CAD patient outcome**

**Study Year (Name of the first Author/Year)**

**Selection**

**Comparability**

**Outcome**

**Total**

Berger. et al. 2004 Medalion. et al. 2004 DeRose. et al. 2005 Schachner. et al. 2005 Fuster. et al. 2006 Salisbury. et al. 2007 Hawkins. et al. 2009 Angouras. et al. 2010 Dagenais. et al. 2010 Konecny. et al. 2010 Nishiyama. et al. 2010 Stelle. et al. 2011 Lazzeri et al. 2011 Efird. et al. 2013 Andell. et al. 2014 Almagro. et al. 2015 Zhang. et al. 2016 Jatene.et al. 2017

Su et al. 2017 Huang et al. 2019 Lin et al. 2019

Tomaniak et al. 2020

Gatta et al. 2021 Wang et al. 2021

4

2

2

2

3

1

1

3

0

2

1

3

2

3

1

4

1

2

1

2

2

2

4

2

2

2

2

2

2

2

2

2

2

2

2

2

2

2

2

2

2

2

2

2

2

2

2

2

3

3

3

3

3

3

3

3

3

3

3

3

3

3

3

3

3

3

3

3

3

3

3

3

9

7

7

7

8

6*

6*

8

5*

7

6*

8

7

8

6*

9

6*

7

6*

7

7

7

9

7

*: Study at high risk of bias, with total score <= 6.

COPD, Chronic Obstructive Pulmonary Disease; NOS, Newcastle–Ottawa Scale.

34


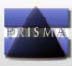


**PRISMA 2020 Checklist**

35

**Section and Topic**

**Item #**

**Checklist item**

**Location where item is reported**

**TITLE**

Title

1

Identify the report as a systematic review.

P1

**ABSTRACT**

Abstract

2

See the PRISMA 2020 for Abstracts checklist.

See abstracts

**INTRODUCTION**

Rationale

3

Describe the rationale for the review in the context of existing knowledge.

P3

Objectives

4

Provide an explicit statement of the objective(s) or question(s) the review addresses.

P3

**METHODS**

Eligibility criteria

5

Specify the inclusion and exclusion criteria for the review and how studies were grouped for the syntheses.

P4

Information sources

6

Specify all databases, registers, websites, organisations, reference lists and other sources searched or consulted to identify studies. Specify the date when each source was last searched or consulted.

P4-5 and Figure 1

Search strategy

7

Present the full search strategies for all databases, registers and websites, including any filters and limits used.

Appendix 1

Selection process

8

Specify the methods used to decide whether a study met the inclusion criteria of the review, including how many reviewers screened each record and each report retrieved, whether they worked independently, and if applicable, details of automation tools used in the process.

P3, P4 and Figure 1

Data collection process

9

Specify the methods used to collect data from reports, including how many reviewers collected data from each report, whether they worked independently, any processes for obtaining or confirming data from study investigators, and if applicable, details of automation tools used in the process.

P5-P6

Data items

10a

List and define all outcomes for which data were sought. Specify whether all results that were compatible with each outcome domain in each study were sought (e.g. for all measures, time points, analyses), and if not, the methods used to decide which results to collect.

P5-6

10b

List and define all other variables for which data were sought (e.g. participant and intervention characteristics, funding sources). Describe any assumptions made about any missing or unclear information.

P5-P6

Study risk of bias assessment

11

Specify the methods used to assess risk of bias in the included studies, including details of the tool(s) used, how many reviewers assessed each study and whether they worked independently, and if applicable, details of automation tools used in the process.

P4-P5

Effect measures

12

Specify for each outcome the effect measure(s) (e.g. risk ratio, mean difference) used in the synthesis or presentation of results.

P5-P6

Synthesis methods

13a

Describe the processes used to decide which studies were eligible for each synthesis (e.g. tabulating the study intervention characteristics and comparing against the planned groups for each synthesis (item #5)).

P5-P6

13b

Describe any methods required to prepare the data for presentation or synthesis, such as handling of missing summary statistics, or data conversions.

P5-P6

13c

Describe any methods used to tabulate or visually display results of individual studies and syntheses.

P5-P6

13d

Describe any methods used to synthesize results and provide a rationale for the choice(s). If meta-analysis was performed, describe the model(s), method(s) to identify the presence and extent of statistical heterogeneity, and software package(s) used.

P5-P6

13e

Describe any methods used to explore possible causes of heterogeneity among study results (e.g. subgroup analysis, meta-regression).

P5-P6

13f

Describe any sensitivity analyses conducted to assess robustness of the synthesized results.

P5-P6

Reporting bias assessment

14

Describe any methods used to assess risk of bias due to missing results in a synthesis (arising from reporting biases).

P5

Certainty assessment

15

Describe any methods used to assess certainty (or confidence) in the body of evidence for an outcome.

P5-P6


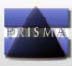


**PRISMA 2020 Checklist**

*From:* Page MJ, McKenzie JE, Bossuyt PM, Boutron I, Hoffmann TC, Mulrow CD, et al. The PRISMA 2020 statement: an updated guideline for reporting systematic reviews. BMJ 2021;372:n71. doi: 10.1136/bmj.n71

For more information, visit: <http://www.prisma-statement.org/>

36

**Section and Topic**

**Item #**

**Checklist item**

**Location where item is reported**

**RESULTS**

Study selection

16a

Describe the results of the search and selection process, from the number of records identified in the search to the number of studies included in the review, ideally using a flow diagram.

P6

16b

Cite studies that might appear to meet the inclusion criteria, but which were excluded, and explain why they were excluded.

-

Study characteristics

17

Cite each included study and present its characteristics.

Table 1 and Table S1.

Risk of bias in studies

18

Present assessments of risk of bias for each included study.

Supplemental Table S7-S8

Results of individual studies

19

For all outcomes, present, for each study: (a) summary statistics for each group (where appropriate) and (b) an effect estimate and its precision (e.g. confidence/credible interval), ideally using structured tables or plots.

Figure S1

Results of syntheses

20a

For each synthesis, briefly summarise the characteristics and risk of bias among contributing studies.

P8

20b

Present results of all statistical syntheses conducted. If meta-analysis was done, present for each the summary estimate and its precision (e.g. confidence/credible interval) and measures of statistical heterogeneity. If comparing groups, describe the direction of the effect.

P6-P9

20c

Present results of all investigations of possible causes of heterogeneity among study results.

P6-P9

20d

Present results of all sensitivity analyses conducted to assess the robustness of the synthesized results.

P6-P9

Reporting biases

21

Present assessments of risk of bias due to missing results (arising from reporting biases) for each synthesis assessed.

P9

Certainty of evidence

22

Present assessments of certainty (or confidence) in the body of evidence for each outcome assessed.

P6-P9

**DISCUSSION**

Discussion

23a

Provide a general interpretation of the results in the context of other evidence.

P10

23b

Discuss any limitations of the evidence included in the review.

P10-P13

23c

Discuss any limitations of the review processes used.

P13

23d

Discuss implications of the results for practice, policy, and future research.

P13

**OTHER INFORMATION**

Registration and protocol

24a

Provide registration information for the review, including register name and registration number, or state that the review was not registered.

P1

24b

Indicate where the review protocol can be accessed, or state that a protocol was not prepared.

P1

24c

Describe and explain any amendments to information provided at registration or in the protocol.

-

Support

25

Describe sources of financial or non-financial support for the review, and the role of the funders or sponsors in the review.

P14

Competing interests

26

Declare any competing interests of review authors.

P14

Availability of data, code and other materials

27

Report which of the following are publicly available and where they can be found: template data collection forms; data extracted from included studies; data used for all analyses; analytic code; any other materials used in the review.

P14
